# Revisiting *Acalypha* medicinal interest: ethnobotany, experimental studies, and the implications of taxonomic misuse pitfalls

**DOI:** 10.3897/phytokeys.270.169087

**Published:** 2026-01-30

**Authors:** Lucía Villaescusa-González, José María Cardiel, Iris Montero-Muñoz, Pablo Muñoz-Rodríguez

**Affiliations:** 1 Department of Biodiversity, Ecology and Evolution, Faculty of Biological Sciences, Universidad Complutense de Madrid, José Antonio Novais, 12, 28040 Madrid, Spain Centro de Investigación en Biodiversidad y Cambio Global (CIBC-UAM), Universidad Autónoma de Madrid Madrid Spain; 2 Department of Biology, Faculty of Sciences, Universidad Autónoma de Madrid, Darwin 2, 28049 Madrid, Spain Department of Biology, Faculty of Sciences, Universidad Autónoma de Madrid Madrid Spain; 3 Centro de Investigación en Biodiversidad y Cambio Global (CIBC-UAM), Universidad Autónoma de Madrid. Darwin 2, 28040 Madrid, Spain Faculty of Biological Sciences, Universidad Complutense de Madrid Madrid Spain; 4 Department of Life Sciences, Faculty of Sciences, Universidad de Alcalá, Alcalá de Henares, 28805 Madrid, Spain Faculty of Sciences, Universidad de Alcalá Madrid Spain

**Keywords:** *
Acalypha
*, ethnobotany, Euphorbiaceae, medicinal plants, pharmacological studies, taxonomic accuracy

## Abstract

*Acalypha* L. (Euphorbiaceae) is a pantropical genus comprising approximately 470 species, many of which have been traditionally used to treat human and animal ailments. Despite its widespread use, the interpretation of ethnobotanical information has been hindered by misidentifications, outdated or incorrect names, and the lack of studies for many species – factors that limit its value for pharmacological research and conservation. Previous efforts to synthesise medicinal knowledge in *Acalypha* have been constrained by limited taxonomic coverage, inconsistent methodologies, and narrow geographic scope. In this study, a comprehensive global review of medicinal uses in *Acalypha* was conducted, based on data retrieved from peer-reviewed literature, scientific databases, historical sources, and other publications. A total of 62 species with reported uses across 55 countries were identified. Uses include applications in human and veterinary medicine, rituals, and as pesticides, while experimental studies reported antibacterial, antifungal, antioxidant, and anti-inflammatory effects. Reported uses were classified as ethnobotanical and/or experimental (*in vitro*, *in vivo*, and *ex vivo*) and standardised following WHO and national disease classification systems, and all scientific names were taxonomically verified. The phylogenetic distribution of medicinal species was assessed using DNA barcode phylogenies. Nearly 25% of the studies reviewed were found to contain at least one taxonomic error, rendering the associated information unreliable and underscoring the need for improved taxonomic rigour and standardisation. This review provides the first standardised, taxonomically validated global synthesis of *Acalypha*’s medicinal knowledge, identifies major knowledge gaps, and offers a foundation for future phytochemical and pharmacological research on this diverse genus.

## Introduction

Throughout history, humans have relied on plants for diverse purposes, particularly for food, clothing, disease prevention, and treatment. Understanding how and why communities use plants is not only valuable from a historical perspective but also critical for identifying new pharmacologically active compounds and establishing conservation priorities (Wheeler 2024). However, many plants with ethnobotanical significance remain poorly studied, especially in the tropics, and the lack of rigorous taxonomic assessment often complicates their accurate identification. Taxonomic uncertainty raises concerns about the reliability of many studies, as misidentifications lead to confusion and even pose risks to human health (Malaspina et al. 2022; Parnmen et al. 2023). Beyond scientific inaccuracies, the misuse of taxonomy also has broader implications, affecting bioprospecting efforts, conservation policies, and the preservation of traditional knowledge. Inaccurate species identification may hinder drug discovery, misinform conservation priorities, and contribute to the erosion of ethnobotanical heritage.

Euphorbiaceae Juss. stands out among the plant families with medicinal use for its high species diversity and global distribution. Within the family, genera such as *Euphorbia* L., *Jatropha* L., and *Ricinus* L. are well known for their medicinal value (Mwine and Van Damme 2011; Islam et al. 2019), but other medicinally relevant genera within the family have received less scientific attention. One such example is *Acalypha* L., the third largest genus in the family, with approximately 470 accepted species and a pantropical distribution (Cardiel et al. 2023). Despite its species richness and documented uses – about one-eighth of *Acalypha* species have been cited in ethnobotanical literature – *Acalypha* remains understudied in comparison to its relatives, and existing studies contain both valuable insights and significant taxonomic inconsistencies, as highlighted in this work. As with many other tropical plant groups, misidentification of *Acalypha* specimens is common, and research frequently includes misapplied or synonymous names and outdated classifications. Understanding the ethnobotanical diversity of *Acalypha* therefore requires a robust and up-to-date taxonomic framework, which remains incomplete for many species.

Over the past two decades, *Acalypha* has received growing taxonomic attention, particularly in South America (Cardiel and Muñoz-Rodríguez 2012; Cardiel et al. 2013a, 2013b, 2023), as well as in Southeast Asia (Sagun et al. 2010), the Western Indian Ocean Region (Montero-Muñoz et al. 2023), and parts of West Africa (Cardiel and Montero-Muñoz 2018). However, geographic coverage remains uneven, with little or no work conducted in many countries, and the majority of species still poorly understood. These gaps hinder the development of a consistent taxonomic framework and reduce the accessibility and usability of existing information (Soladoye et al. 2008). In parallel, attempts to review the genus’s medicinal uses have been constrained by heterogeneous data, a lack of taxonomic verification, and limited source coverage, further complicating systematic comparison.

In this study, we present a critical synthesis of ethnobotanical knowledge and experimental research on *Acalypha*. We compile and categorise all reported uses within a rigorous taxonomic framework. By integrating data from diverse sources, standardising reported uses, and correcting taxonomic inconsistencies, we aim to provide a clearer and more reliable foundation for future research. Several *Acalypha* species show promising medicinal properties, underscoring the need for a systematic reassessment of their ethnobotanical and pharmacological potential.

## Methods

### Data collection and classification

Data collection

We conducted a systematic literature review using major scientific databases and specialised repositories (Google Scholar, PubMed, Biodiversity Heritage Library, HathiTrust), applying various keyword combinations (e.g. ‘*Acalypha* ethnobotany’, ‘*Acalypha* medicinal properties’) in multiple languages and refining results with Boolean operators. To improve coverage, we also examined the reference lists of retrieved studies and repeated the search iteratively until reaching saturation – that is, when no new relevant articles emerged. Although herbarium specimens often include annotations on medicinal use, this review focuses exclusively on published bibliographic sources for practical reasons.

#### Data classification

A key limitation in previous studies was the lack of standardised classification, which hinders meaningful comparisons. To address this, we compiled all information into a searchable database (Suppl. material 1) and classified the data into five major categories: 1) ethnobotanical, 2) *in vitro* experiments, 3) *in vivo* experiments, 4) *ex vivo* experiments, and 5) phytochemical studies. Each category was further subdivided, and we recorded the following parameters consistently across study types:

- Plant part used: roots, stems, stem bark, branches, leaves, flowers, fruits, or the whole plant.
- Plant use: categorised by purpose (human or veterinary medicine, plaguicidal, and ritual uses). For medicinal uses, we standardised specific ailments by disease type or symptoms treated.
- Location: for experimental studies, the collection site; for ethnobotanical studies, the country and, when available, the human group or locality.

#### Data organisation

##### Ethnobotanical studies

We compiled all recorded traditional uses of *Acalypha* species from ethnobotanical sources, experimental studies, and relevant secondary literature. In addition to standardised categories, we recorded the following specific features:

- General use categories: human medicine (treatment of human diseases), veterinary medicine (treatment of non-human animal diseases), plaguicidal use targeting insect vectors of human diseases (excluding agricultural pesticides), and ritual uses (e.g. “insanity and possession” or “magical properties”), where the exact purpose of use is often unclear.
- Use method: preparation techniques, including combinations with other plants, which may influence the concentration, efficacy, and therapeutic effect of active compounds.
- Disease classification: standardised following the *International Classification of Diseases for Mortality and Morbidity Statistics* (World Health Organization 2022) and the *Spanish Inventory of Traditional Knowledge related to Biodiversity* (Pardo de Santayana et al. 2014) (Table 1, Suppl. material 1). We applied this classification system uniformly to both ethnobotanical and experimental studies to enable direct comparison.

##### *In vitro* studies

We classified studies testing *Acalypha* extracts in cellular models (excluding living animals) according to the following criteria:

**Table 1.** Disease classification system used in this study. For a detailed comparison with the reference frameworks (Pardo de Santayana et al. 2014; World Health Organization 2022), see Suppl. material 1. No *Acalypha* species were found to be used in the treatment of mental disorders. Sexually transmitted diseases (STDs) are treated as a separate category from infectious and parasitic diseases.

Classification used in this study		Specifications
Primary classification	Subcategories	
Cardiovascular system	-	Diseases related to the heart and blood vessels, such as hypertension, strokes, etc.
Digestive tract	-	Conditions affecting the stomach, liver, intestines, and related organs.
Urinary system	-	Diseases of the kidneys, bladder, and urinary tract (e.g. “diuretic” and “urinary complaints”).
Pregnancy, childbirth, and puerperium	-	Conditions occurring during pregnancy, labour, delivery, and the postpartum period, such as “postpartum pain” or “stop bleeding during pregnancy”.
Perinatal period	-	Diseases affecting the foetus or newborn (e.g. perinatal infections).
Reproductive system	Abortifacient	Use as agents to induce abortion or interrupt pregnancy.
	Fertility	Related to enhancing or impairing fertility, including treatments for infertility or as contraceptives.
	Others	Other reproductive issues not covered by the above.
Gynaecological problems	-	Disorders specific to the female reproductive system (e.g. “menstrual pain” and “regulate menstruation”).
Respiratory system	-	Diseases of the lungs and airways, including asthma, bronchitis, coughs, etc.
Endocrine-metabolic system	-	Conditions related to hormonal imbalances and metabolic disorders, such as diabetes and thyroid problems.
Immune system	-	Diseases related to immune function.
Locomotor system	-	Disorders involving muscles, bones, and joints (e.g. “arthritis” and “rheumatism”).
Skin	-	Dermatological conditions, both specific and general (e.g. scabies, pimples, itching).
Mental disorders	-	Disorders related to psychological or psychiatric conditions such as depression, anxiety, or other forms of mental distress or behavioural disturbance. Although included in the classification system for completeness, no records referring to this group were identified in the data reviewed.
Nervous system	-	Includes records associated with neurological conditions or imbalances (e.g. epilepsy, convulsions).
Eye problems	-	Disorders affecting the eyes, such as “conjunctivitis” or “sore eyes”.
Ear problems	-	Diseases or symptoms related to the ears, such as “earache” or ear infections.
Mouth problems	-	Conditions affecting the mouth (e.g. “toothache”, “tooth decay”, “gum disease”).
Infectious or parasitic diseases	-	Bacterial, viral, fungal, and parasitic infections not categorised elsewhere.
STDs	-	Sexually transmitted diseases (e.g. syphilis and gonorrhoea) are treated as a separate category from general infections.
Neoplasm	-	Tumours or abnormal tissue growths, benign or malignant.
Symptoms	Pain-killer	Records referring to isolated symptoms or general sensations of discomfort. These cases are often reported without a clear underlying diagnosis, and the symptom itself is treated as a sufficient basis for classification. Subcategories are organised according to the type of manifestation, allowing for the inclusion of records that reflect common patterns of suffering even when their aetiology remains unspecified.
	Diarrhoea	
	Fever	
	Inflammation	
	Dizziness	
	Others	
Injuries	Bites and stings	Records referring to external harm or damage, typically acute and visible, such as wounds, burns, or stings. Subcategories reflect the type of injury rather than the anatomical system affected.
	Wounds	
	Ulcers, sores	
	Burns	
	Others	
Poisoning	-	Uses in cases of suspected intoxication.
Lymphatic system	-	Both localised and systemic manifestations of lymph-related dysfunction (e.g. lymphoid swellings, elephantiasis).
Others	-	Records that do not clearly fit into any of the predefined categories. This may include ambiguous or broad health-related indications that could not be confidently assigned elsewhere.

- Extract and assay type: we documented solvents used (e.g. acetone, methanol, ethyl acetate) and assay methods (e.g. disc diffusion method, dilution method), as these can influence the observed biological effects.
- Effect: we standardised the biological activity of each plant extract (e.g. antibacterial, antifungal) using the disease classification applied throughout this review.
- Cell lines: we documented the specific cell lines used, as they are critical for interpreting the potential therapeutic applications of each extract.

##### *In vivo* and *ex vivo* studies

For studies conducted on living animals (*in vivo* tests) or isolated tissues (*ex vivo* tests), we applied the same methodology as for *in vitro* studies, with the primary distinction that the experiments were conducted in whole organisms or tissues rather than in cell cultures.

### Phytochemical studies

We documented studies that conducted phytochemical screenings as part of experimental research, as well as those focused exclusively on identifying compounds potentially responsible for medicinal activity. Although a detailed classification of phytochemical compounds is beyond the scope of this review, our dataset provides a valuable foundation for future research on bioactive compounds in the genus.

### Taxonomic and geographic data verification

#### Taxonomic name verification

We carried out a comprehensive taxonomic and nomenclatural review of all *Acalypha* species names cited in the literature reviewed. We verified all names according to the taxonomic framework current at the time of this assessment (2025), following the most authoritative and up-to-date sources available. We classified each name into one of three categories:

1. Correctly recorded names, i.e. names that match current accepted nomenclature, including author citation, orthography, and usage.
2. Names lacking author citation but attributable to a single validly published name, i.e. cases where the omission does not create ambiguity because only one author has validly published the name; these were considered tractable.
3. Incorrectly recorded names, defined as meeting one or more of the following criteria:

1. Synonyms, i.e. names not accepted under current taxonomic criteria. Although synonyms are not considered errors, they were quantified here to reflect outdated nomenclature and to provide a reference framework for future taxonomic updates.
2. Ambiguous names lacking author citation, where the epithet has been validly published by more than one author, rendering the name unassignable without the full citation.
3. Names not in accordance with the rules of the ICN, including *nomina illegitima* or names not validly published.
4. Undetermined names, such as *Acalypha* sp., which lack species-level resolution and are thus considered incomplete.
5. Non-existent names, i.e. names based on misattributed authorship or orthographic errors that do not correspond to any validly published names.

We corrected all names using taxonomic literature published by ourselves and others, as well as resources such as the *Acalypha Taxonomic Information System* (ATIS; Cardiel et al. 2025), developed and maintained by us, and *Plants of the World Online* (RBG Kew 2025). When authorship was ambiguous, we used species distribution data to infer the most likely identity of the plant. However, in a few cases, limited geographic information prevented us from resolving the ambiguity.

Although *Acalypha* sp. is a properly formatted designation, it lacks species-level specificity, and we treated it as an incompletely recorded taxon. We also flagged and corrected “non-existent” names based on incorrect author attribution. We included secondary sources such as review papers in our assessment, as these often contribute to the spread of taxonomic errors.

#### Vernacular name documentation

We recorded common names where provided, specifying the places, human groups, and languages in which they are used. This facilitates linking vernacular names with their corresponding scientific taxa, aiding future research.

#### Geographic data collection and mapping

We categorised location data from ethnobotanical and experimental sources. Standardising ethnobotanical study locations was challenging due to varying specificity levels – from vague regional references to highly detailed locality names – and almost always without geographical coordinates. In contrast, experimental studies generally provide clear collection site information.

For cases lacking precise coordinates, we estimated locations using the centroid of the smallest identifiable geographic area.

We generated all maps using QGIS, with map layers sourced from Natural Earth. We used species distribution data from ATIS, since these have been verified by us as taxonomic experts.

#### Evolutionary relationships and phylogenetic exploration

We aimed to explore whether *Acalypha* species with recorded medicinal or ethnobotanical uses are phylogenetically clustered or dispersed across the genus, as this may inform future research by identifying clades with shared bioactive traits. To investigate this, we mapped all medicinally relevant species onto a phylogeny reconstructed from sequence data published by Levin et al. (2022). A phylogenetic signal in medicinal use (evident through clustering) could suggest that related species share similar phytochemical profiles. In contrast, a scattered distribution might indicate that such properties are more strongly shaped by ecological factors than by shared ancestry.

We focused on the nuclear ribosomal internal transcribed spacer (*nrITS*), which provides limited but sufficient resolution for comparative purposes within *Acalypha*. We retrieved 155 *nrITS* sequences from GenBank, including four outgroup taxa: *Bernardia viridis*, *Erythrococca natalensis*, *Micrococca capensis*, and *Mareya micrantha* (Suppl. material 2). We aligned the sequences using MAFFT v.7.310 (Katoh and Standley 2013, 2016) and filtered the alignment using -automated1 (Capella-Gutiérrez et al. 2009). We then inferred an approximate maximum-likelihood phylogeny using IQ-TREE v.2.4.1 (Minh et al. 2020), with automatic model selection via ModelFinder (Kalyaanamoorthy et al. 2017) and 1,000 ultrafast bootstrap replicates. The SYM+I+G4 model was selected based on the Bayesian Information Criterion. In the resulting phylogeny, we collapsed all nodes with less than 60% support into polytomies.

## Results

### Literature coverage and geographic distribution

We compiled information from 216 published works, including peer-reviewed articles, books, and other documents published between 1816 and 2024. Of these, 137 studies contain ethnobotanical information, and 100 included experimental data, with several contributing to both categories.

We documented *Acalypha* species with ethnomedicinal uses in 55 countries (Table 2), primarily in tropical regions. Despite high species richness in some countries – such as Mexico with 92 species or Brazil and Madagascar with 41 species each (Cardiel et al. 2025) – medicinal research has focused on a limited subset of taxa, often repeatedly across studies. The countries with the highest number of medicinal species reported are India (9 species), Brazil (8 species), Indonesia (6 species), and Papua New Guinea (6 species). In addition, 16 species with reported traditional medicinal uses lacked specific geographic records: *A. allenii* Hutch., *A. amentacea* Roxb., *A. brachystachya* Hornem., *A. decaryana* Leandri, *A. engleri* Pax, *A. emirnensis* Baill., *A. filiformis* Poir., *A. guatemalensis* Pax & K.Hoffm., *A. peduncularis* Meisn. ex C.Krauss, *A. platyphylla* Müll.Arg., *A. polymorpha* Müll.Arg., *A. integrifolia* Willd., *A. radula* Baker, *A. segetalis* Müll.Arg., *A. spachiana* Baill., and *A. volkensii* Pax.

**Table 2.** Geographical data on ethnomedicinal *Acalypha* species by country. The table includes the number of accepted *Acalypha* species per country, the number of those with recorded ethnomedicinal uses, and the number of relevant studies. Of the 55 countries documented, only 53 are shown here. Angola and Zambia are excluded due to the absence of explicit location data, although some species are currently restricted to those countries and thus included in our estimations. Only countries with explicitly recorded data are included.

Country	N° accepted *Acalypha* species	N° accepted *Acalypha* species reported in ethnomedicinal studies	N° studies with *Acalypha* ethnomedicinal information
Argentina	17	1	1
Australia	9	1	3
Bangladesh	1	2	5
Belize	18	1	1
Brazil	41	8	2
Burundi	13	1	2
Cambodia	2	2	1
Central African Republic	5	1	1
China	18	2	4
Comoras	7	1	1
Cuba	19	1	1
Djibouti	2	2	5
Democratic Republic of the Congo	26	1	2
Ethiopia	15	2	3
Fiji	6	2	4
Gabon	2	1	1
Ghana	8	1	1
Guatemala	31	1	7
Honduras	14	1	1
India	12	9	39
Indonesia	20	6	6
Ivory Coast	5	1	1
Kenya	19	3	6
Madagascar	41	2	3
Malawi	21	1	1
Malaysia	5	3	5
Mauritius	5	2	3
Mexico	92	5	15
Mozambique	16	1	1
Namibia	8	1	1
Nepal	3	1	1
Nigeria	9	4	16
Oman	1	1	1
Papua New Guinea	18	7	6
Peru	29	1	2
Philippines	8	2	1
Reunion	4	2	1
Rwanda	9	1	1
Samoa	5	1	1
Senegal	5	1	2
Seychelles	2	1	1
Solomon Islands	5	1	1
South Africa	19	2	5
Sri Lanka	2	1	1
Tanzania	27	3	8
Thailand	10	3	5
Uganda	17	2	6
United States of America	11	1	4
Vanuatu	1	1	2
Venezuela	17	1	1
Vietnam	7	3	7
Zimbabwe	15	1	2
Total number of accepted ethnomedicinal species without specific geographical record	15	Total number of accepted species with ethnobotanical uses	59

**Figure 1.**
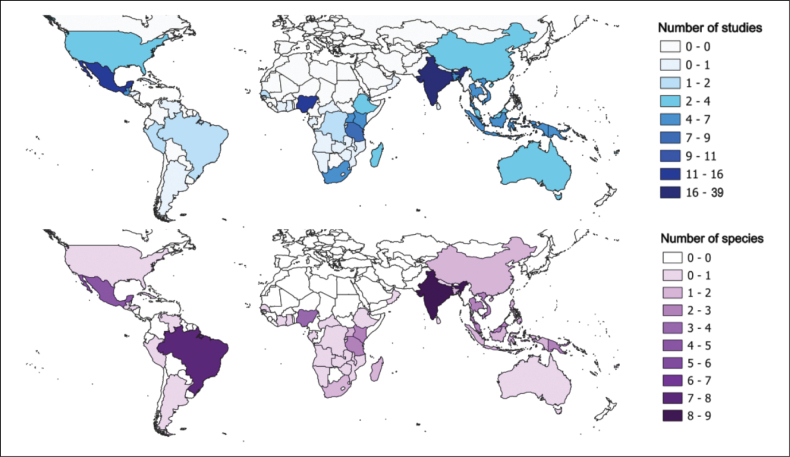
Geographical distribution of *Acalypha* ethnobotanical knowledge. Top: number of studies with ethnobotanical information per country. Bottom: number of species with reported medicinal uses per country. Darker colours indicate higher values in both cases.

Research efforts are heavily concentrated in a few countries, notably India (39 studies), Nigeria (16 studies), and Mexico (15 studies). In contrast, several countries with high *Acalypha* species richness – such as Angola, Brazil, the Democratic Republic of the Congo, Madagascar, Peru, and Tanzania – are severely underrepresented. In many cases, *Acalypha* is only mentioned incidentally, with just a single study available for the entire country, as seen in Argentina, the Central African Republic, or Oman (Fig. 1).

### Medicinal species and disease types

**Figure 2.**
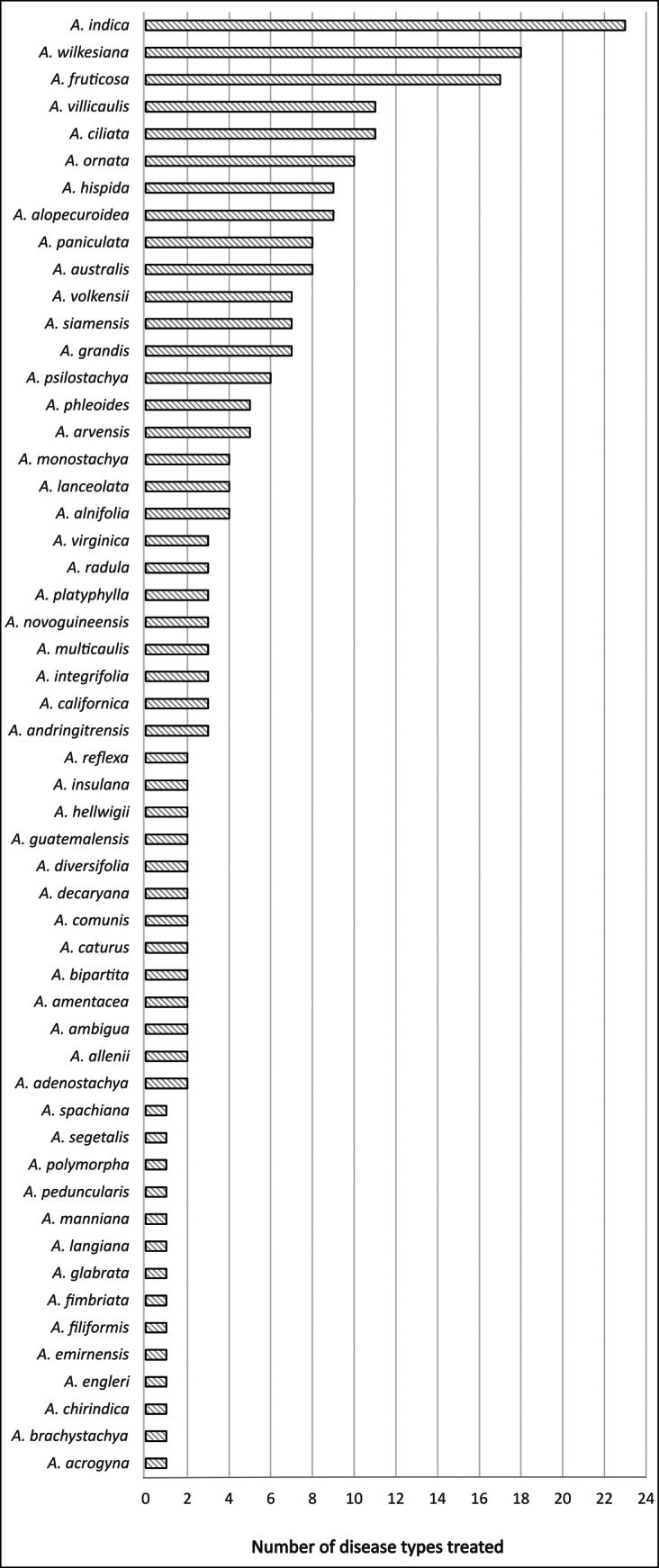
Number of disease types reported for each medicinal *Acalypha* species. Species for which no specific disease type was reported (*A. accedens*, *A. amblyodonta*, *A. cuneata*, *A. glandulifolia*, *A. gracilis*, *A. neptunica*, and *A. poiretii*), or whose use pertains exclusively to non-medicinal categories (pesticides and rituals: *A. echinus*, *A. gaumeri*, and *A. neptunica*), are not shown.

**Figure 3.**
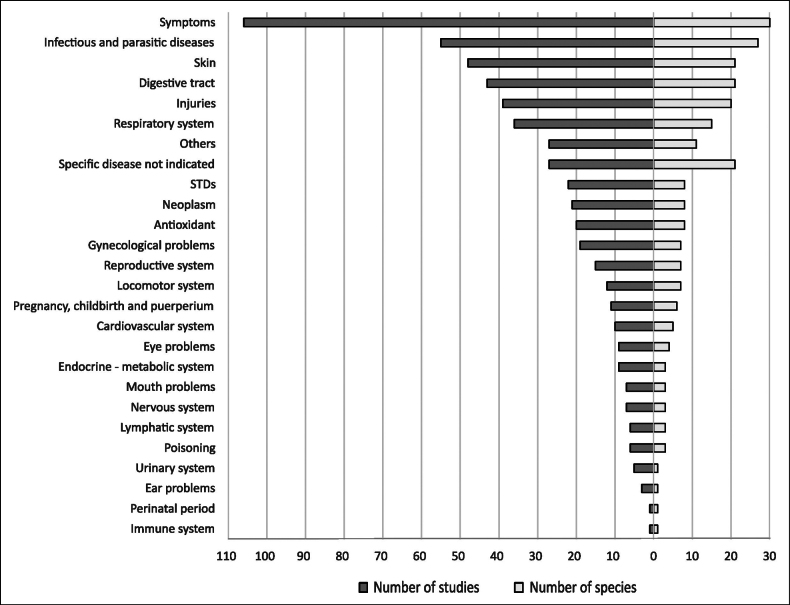
Total number of *Acalypha* studies and species associated with each disease category, sorted by frequency.

We identified 62 *Acalypha* species with documented medicinal uses (Table 3). The species reported to treat the highest number of disease types include *A. indica* (used for treating 23 diseases), *A. wilkesiana* Müll.Arg. (18), *A. fruticosa* Forssk. (17), *A. ciliata* Forssk. and *A. villicaulis* Hochst. ex A.Rich. (11 each), and *A. ornata* Hochst. ex A.Rich. (nine) (Fig. 2). With few exceptions, most species have been cited in only a handful of studies (Suppl. materials 1, 3). The most frequently reported disease categories are ‘symptoms’, ‘infectious diseases’, ‘skin’, ‘digestive tract’, and ‘injuries’, which also involved the highest number of species (Fig. 3).

**Table 3.** Checklist of *Acalypha* species with documented medicinal uses. An asterisk (*) indicates species for which no location information was available.

Species		Ethnobotanical information	Experimental information
1	*A. accedens* Müll.Arg.	X	
2	*A. acrogyna* Pax	X	
3	*A. adenostachya* Müll.Arg.	X	
4	*A. allenii* Hutch.	X*	
5	*A. alnifolia* J.G.Klein ex Willd.	X	X
6	*A. alopecuroidea* Jacq.	X	X
7	*A. ambigua* Pax	X	
8	*A. amblyodonta* Müll.Arg.	X	
9	*A. amentacea* Roxb.	X*	
10	*A. andringitrensis* Leandri	X	
11	*A. arvensis* Poepp.	X	
12	*A. australis* L.	X	X
13	*A. bipartita* Müll.Arg.	X	X
14	*A. brachystachya* Hornem.	X*	
15	*A. californica* Benth.	X	X
16	*A. caturus* Blume	X	
17	*A. chirindica* S.Moore	X	
18	*A. ciliata* Forssk.	X	X
19	*A. communis* Müll.Arg.	X	X
20	*A. cuneata* Poepp.	X	
21	*A. decaryana* Leandri	X*	
22	*A. diversifolia* Jacq.		X
23	*A. echinus* Pax & K.Hoffm.	X	
24	*A. engleri* Pax	X*	
25	*A. emirnensis* Baill.	X*	
26	*A. fimbriata* Schumach. & Thonn.		X
27	*A. filiformis* Poir.	X*	
28	*A. fruticosa* Forssk.	X	X
29	*A. gaumeri* Pax & K.Hoffm.		X
30	*A. glabrata* Thunb.	X	
31	*A. glandulifolia* Buchinger & Meisn. ex C.Krauss	X	
32	*A. gracilis* Spreng.	X	
33	*A. grandis* Benth.	X	X
34	*A. guatemalensis* Pax & K.Hoffm.	X*	X*
35	*A. hellwigii* Warb.	X	
36	*A. hispida* Burm.f.	X	X
37	*A. indica* L.	X	X
38	*A. insulana* Müll.Arg.	X	
39	*A. integrifolia* Willd.	X	X*
40	*A. lanceolata* Willd.	X	
41	*A. langiana* Müll.Arg.	X	X
42	*A. manniana* Müll.Arg.	X	
43	*A. monostachya* Cav	X	X
44	*A. multicaulis* Müll.Arg.	X	
45	*A. neptunica* Müll.Arg.	X	
46	*A. novoguineensis* Warb.	X	
47	*A. ornata* Hochst. ex A.Rich.	X	X
48	*A. paniculata* Miq.	X	X
49	*A. peduncularis* Meisn. ex C.Krauss	X*	
50	*A. phleoides* Cav.	X	X
51	*A. platyphylla* Müll.Arg.	X*	X
52	*A. poiretii* Spreng.	X	
53	*A. polymorpha* Müll.Arg.	X*	
54	*A. psilostachya* Hochst. ex A.Rich.	X	
55	*A. radula* Baker	X*	
56	*A. reflexa* Müll.Arg.	X	X
57	*A. segetalis* Müll.Arg.	X*	X
58	*A. siamensis* Oliv ex. Gage	X	X
59	*A. spachiana* Baill.	X*	
60	*A. villicaulis* Hochst. ex A.Rich.	X	
61	*A. volkensii* Pax	X*	
62	*A. wilkesiana* Müll.Arg.	X	X

Of the 62 species reviewed, 59 have recorded ethnobotanical uses. Of these, 23 have also been included in at least one experimental study, while 36 have not been studied experimentally. Three additional species (*A. diversifolia* Jacq., *A. gaumeri* Pax & K.Hoffm., and *A. fimbriata* Schumach. & Thonn.) have been studied in the laboratory despite lacking known ethnobotanical records (Table 3). However, the boundary between ethnobotanical and experimental studies is often blurred, as many experimental papers also include ethnobotanical information, and vice versa.

Among the 137 studies with ethnobotanical data, we classified uses into four main categories: ‘human medicine’ (125 studies), ‘veterinary medicine’ (16), ‘rituals’ (2), and ‘pesticide’ (3) (Table 4). Several species appeared in multiple use categories and had more than one use per category (Suppl. materials 1, 3).

**Table 4.** Summary of the number of *Acalypha* species and studies by general study type. Note that categories are not mutually exclusive, as individual studies may include multiple approaches.

	General use/type of study	Number of species	Number of studies
Studies with ethnobotanical information	Human medicine	58	125
	Veterinary medicine	4	16
	Rituals	7	2
	Plaguicide	5	3
	Category not indicated	1	1
Studies with experimental information	*In vitro*	19	60
	*Ex vivo*	3	6
	*In vivo*	14	42
	Total	62	216

**Figure 4.**
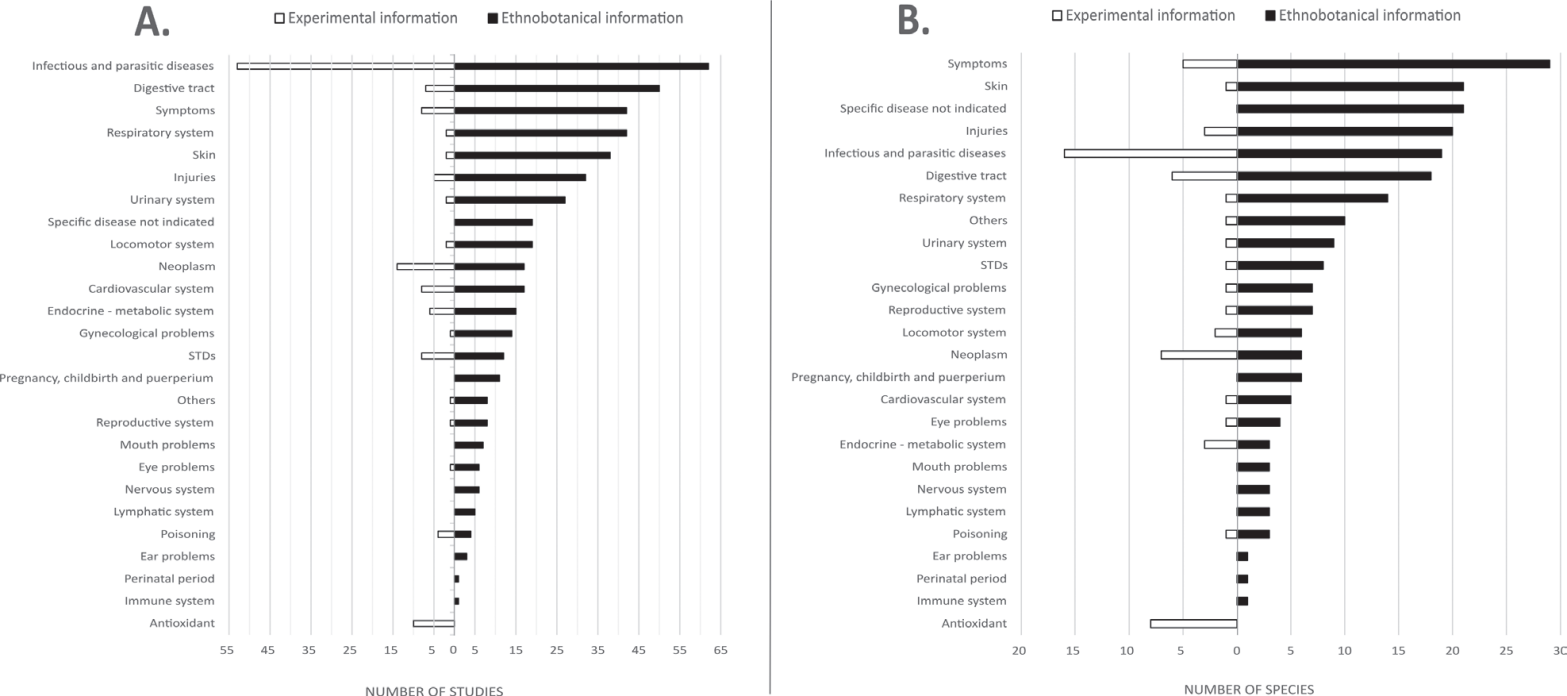
Comparative analysis of *Acalypha* medicinal use, showing the number of studies (A) and species (B) reported in ethnobotanical and experimental data for each disease category.

In human medicine, *Acalypha* species have been used to treat all disease categories listed in Table 1, except mental disorders. The five most frequently cited categories are ‘infectious and parasitic diseases’, ‘digestive tract’, ‘symptoms’, ‘respiratory system’, and ‘skin’. In general, the disease categories with the highest number of studies also correspond to those involving the greatest number of species (Fig. 4).

We identified 100 studies with experimental data. Among the 23 species studied experimentally, only three (*A. fruticosa*, *A. phleoides*, and *A. wilkesiana*) have been tested *in vitro*, *ex vivo*, and *in vivo*. Five species have been included in both *in vitro* and *in vivo* experiments (but not *ex vivo*), 11 species have been studied exclusively *in vitro*, and four exclusively *in vivo*. Phytochemical information was inconsistently reported and will be treated in a separate synthesis currently underway (Villaescusa-González et al. in prep.).

Most experimental studies target ‘infectious and parasitic diseases’ and, to a lesser extent, ‘neoplasms’ and ‘antioxidant’ activity. The number of species studied follows a similar trend, while other disease categories are rarely represented (Fig. 4).

### Phylogenetic relationships and experimental focus

**Figure 5.**
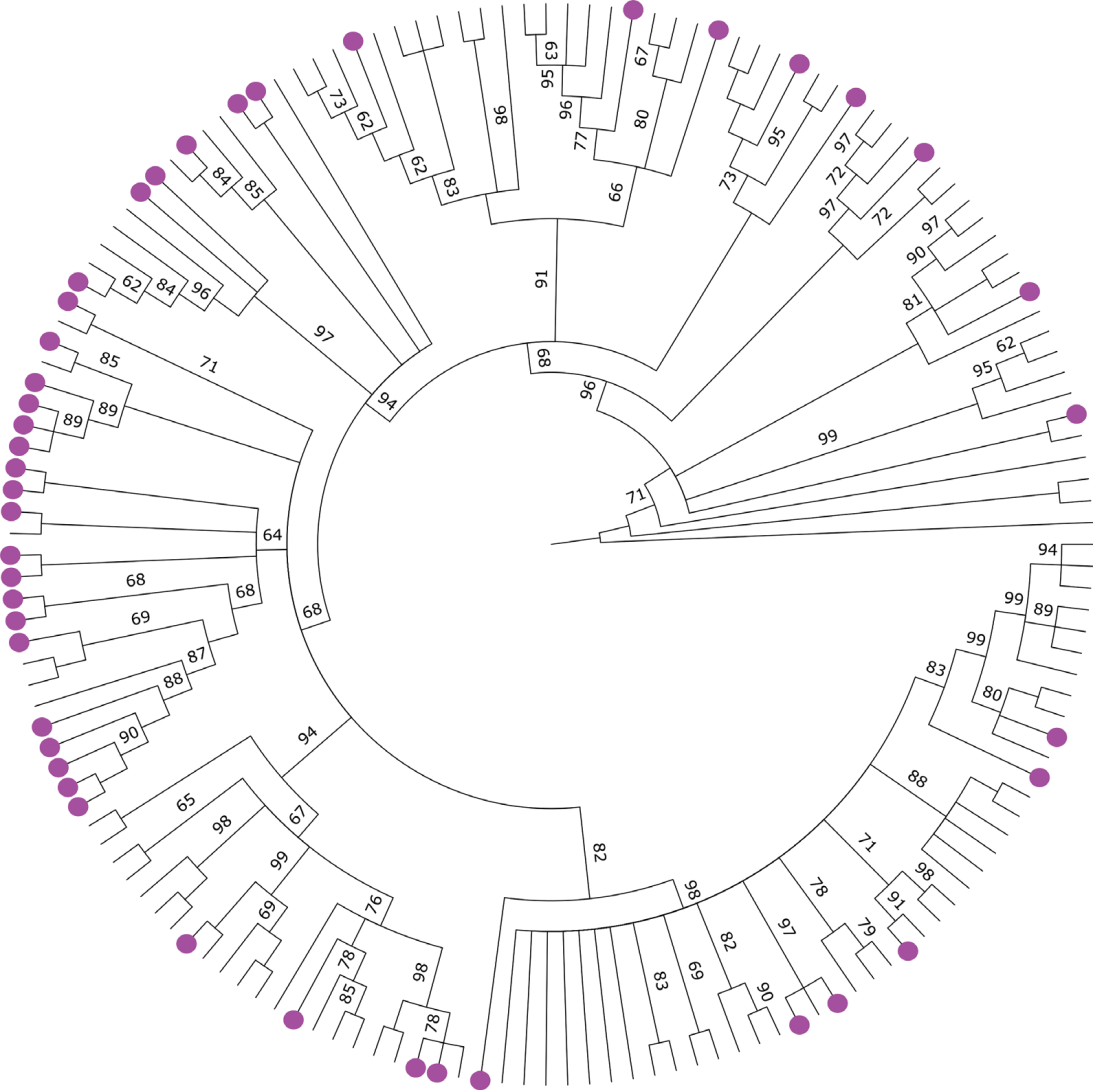
Phylogenetic distribution of medicinal *Acalypha* species (purple dots). The tree is a maximum-likelihood phylogeny inferred from 155 *nrITS* sequences. Nodes lacking a reported support value have 100% bootstrap support. See expanded phylogeny with taxon names in Suppl. material 2.

To examine evolutionary relationships, we inferred a phylogenetic tree based on 155 sequences, 46 of which correspond to species included in this review. These species are distributed throughout the phylogeny, with no evident clustering except perhaps in subgenus *Acalypha* (Fig. 5). However, this pattern should be regarded as preliminary, since the available molecular data represent only c. 30% of the nearly 470 species of *Acalypha* – and sampling in subgenus *Acalypha*, where most species with medicinal properties belong, is very limited.

### Taxonomic reliability

**Figure 6.**
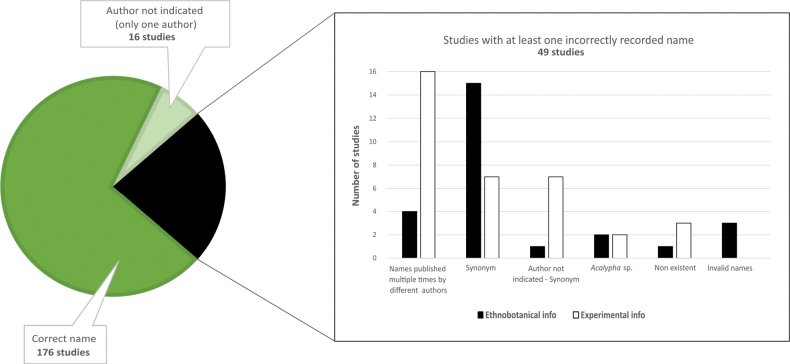
Taxonomic and nomenclatural uncertainties appear in c. 25% of *Acalypha* medicinal records. An incorrectly recorded name is defined here as one meeting any of the following criteria: (1) a synonym; (2) lacking author citation for names published multiple times by different authors; (3) a combination of synonymy and missing author citation; (4) an incorrect name, including either *nomina illegitima* or names invalidly published; (5) genus-only identification (*Acalypha* sp.); or (6) a non-existent name due to incorrectly attributed authorship.

Our comprehensive taxonomic and nomenclatural review revealed that nearly 25% of the articles reviewed contained at least one incorrectly written species name. Specifically, we found 18 studies citing names now considered synonyms. Although such names were likely valid at the time of publication, we quantified them to reflect outdated nomenclature and to provide a reference framework for future taxonomic updates. We also found 20 studies lacking author citations that could not be assigned, eight without any author citation, four that did not specify the species, four with non-existent names, and three with formally incorrect names (Fig. 6).

Taken together, these findings reveal a fragmented and uneven picture of medicinal knowledge in *Acalypha*. The distribution of studies is biased across species, countries, and disease categories, and widespread taxonomic inaccuracies further hinder interpretation. We explore these gaps and inconsistencies in detail in the following section.

## Discussion

Despite being a large and diverse pantropical genus with over 470 recognised species, only 62 species of *Acalypha* have documented medicinal uses, and just 23 have been evaluated through experimental studies. Our phylogenetic analysis, which includes approximately one-third of the genus, shows that medicinally relevant species are scattered across the tree (Fig. 1), suggesting that unexplored pharmacological potential may be widespread. This pattern highlights significant opportunities for discovery across multiple clades.

Many *Acalypha* species remain poorly known, often collected only once or a few times, and have not been studied since their original description. These underexplored taxa may hold valuable medicinal properties and should be prioritised in future research alongside species that have already shown pharmacological potential. Our review offers a structured baseline to guide such work, combining taxonomic rigour with a standardised synthesis of ethnobotanical and experimental knowledge.

In the following sections, we examine key geographic, taxonomic, methodological, and thematic gaps and biases affecting current knowledge of *Acalypha*’s medicinal uses and outline priorities for a more integrative and comprehensive approach moving forward. Detailed information for all species and uses is provided in Suppl. materials 1, 3, and the ATIS online platform (https://acalypha.es).

### Geographic biases in ethnobotanical and experimental research

Ethnobotanical information on *Acalypha* has been documented from 55 countries, although research efforts are disproportionately concentrated in a few, notably India (39 studies), Nigeria (16 studies), and Mexico (15 studies). In contrast, several regions with high *Acalypha* species richness are severely underrepresented. This geographic bias is even more pronounced in experimental research, which is largely confined to Nigeria and India. As a result, many regions rich in *Acalypha* species remain virtually unexplored from a pharmacological perspective, representing a significant gap in current knowledge.

Notably, some of the most widely distributed species also emerge as the most studied and versatile in terms of reported medicinal uses. For instance, *Acalypha indica*, a broadly distributed palaeotropical weedy species, is reported to treat up to 23 disease types, likely reflecting its widespread accessibility. *Acalypha wilkesiana* and *A. fruticosa* follow with 18 and 17 disease types, respectively (Fig. 2). This pattern suggests that wider geographic distribution and weedy habit may increase the likelihood of both ethnobotanical documentation and experimental investigation – a pattern already suggested in previous studies in Ecuador (Hart et al. 2017), Spain (Mateo-Martín et al. 2023), and the Balkan Mountains (Alrhmoun et al. 2024), among others. However, many other *Acalypha* species with wide distributions or ecological significance, such as *A. paniculata* Miq. or *A. diversifolia*, remain understudied, indicating that distribution alone does not determine research attention.

### Methodological and thematic biases

Beyond geographical limitations, available studies on *Acalypha* are affected by methodological and thematic biases rooted in the inherent differences between ethnobotanical and experimental approaches. Ethnobotanical studies typically document treatment of symptoms rather than clinically defined diseases, reflecting traditional knowledge systems that focus on observable signs rather than clinical diagnostic categories (Staub et al. 2015). This approach, while culturally valid, complicates direct comparisons with experimental studies, which usually target well-defined pathologies (Fig. 3).

Experimental research, in contrast, tends to concentrate on infectious and parasitic diseases – and to a lesser extent on cancer (classified under neoplasms) – likely because *in vitro* models using bacteria, fungi, and parasites are more affordable and logistically feasible than *in vivo* studies that require specialised infrastructure, trained personnel, and regulatory compliance (Houghton et al. 2007). Similarly, additional funding interest in oncology likely amplifies the focus on neoplasms. In contrast, other disease categories, especially those requiring more complex experimental models, remain underrepresented.

Ethnobotanical research, by avoiding heavy infrastructure or funding requirements, often captures a broader array of medicinal uses across a wider spectrum of health conditions (Fig. 4). However, reliability varies depending on accurate species identification and the depth and continuity of local knowledge.

Finally, heterogeneity in methods and reporting remains a challenge. Studies range widely in scope, terminology, and methodological rigour, making synthesis difficult. In addition, many experimental studies test *Acalypha* extracts in combination with other plants or use diverse extraction methods (e.g. ethanol, methanol, etc.), making it challenging to isolate the specific effects of *Acalypha*. While some degree of methodological variability is inevitable, there is growing consensus on the need for improved standardisation – otherwise, the lack of standardisation contributes to the fragmentation of existing knowledge and limits the integration of findings across studies (De Albuquerque and Hanazaki 2009).

### Data heterogeneity and limitations of previous studies

A major challenge in assessing the medicinal potential of *Acalypha* lies in the heterogeneity of available data and the limitations of previous reviews. Most existing reviews have focused on individual species – most notably *Acalypha indica* – or have been restricted to either ethnobotanical or experimental data. A representative example is the review by Zahidin et al. (2017), which provides a useful overview of experimental studies on *A. indica* but does not incorporate ethnobotanical information.

Until this work, Seebaluck et al. (2015) was the only published attempt at a global review integrating both ethnobotanical and experimental data across *Acalypha* species. While the work by Seebaluck and colleagues was a valuable contribution, it presents several limitations. First, the number of studies reviewed was relatively small: in our assessment, we identified 158 studies that were entirely or partially omitted from Seebaluck et al.’s compilation – of which only 16 were published after their review and a further eight in the same year. In addition, the taxonomic treatment was inconsistent, and the information was not standardised. This highlights a gap not only in available data but also in how that information has been historically selected and synthesised, suggesting that some relevant contributions may have been underrepresented.

Beyond the scope of specific reviews, a broader issue is the marked variability in the structure, focus, and terminology used across ethnobotanical and experimental studies. These differences hinder direct comparison and systematic analyses. In particular, the absence of unified standards for classifying diseases, uses, and pharmacological assays contributes to fragmented and often incomparable datasets. Although language could in theory act as a barrier to accessing relevant literature, we found that the number of pertinent publications in languages other than English – such as French, Spanish, or Chinese – is relatively small. Therefore, we do not consider language bias to be a significant factor affecting the availability or accessibility of information included in this review.

### Taxonomic shortcomings and consequences

A persistent and critical limitation in both ethnobotanical and pharmacological studies on *Acalypha* is the lack of taxonomic rigour. We identified 49 published studies – approximately 25% of all references analysed – in which at least one species name was misapplied, misspelled, or incompletely cited (Fig. 6). While some of these issues, such as outdated synonyms, can be resolved through standard taxonomic verification, others pose more serious challenges. Missing author citations, incorrect species attributions, or ambiguous names complicate efforts to accurately link data to the correct taxa. This problem is particularly concerning given that secondary sources often perpetuate these errors, amplifying taxonomic inaccuracies across successive publications. Furthermore, the majority of studies fail to provide or cite voucher specimens, making independent verification of identifications virtually impossible. The absence of such essential documentation severely limits reproducibility and precludes later taxonomic reassessment as knowledge advances. Although we noted whether vouchers were cited when reported, we did not systematically record this information across all studies, as it lay beyond the scope of the present review. This aspect is being addressed in our ongoing synthesis of *Acalypha* phytochemical research (Villaescusa-González et al. in prep.), where voucher documentation is being tracked comprehensively.

These inconsistencies are often based on the unexamined assumption that species identifications are correct – a premise that our review shows is frequently unfounded. For instance, *Acalypha alnifolia* J.G.Klein ex Willd., a species endemic to India, was reportedly collected in Nigeria (Noumedem et al. 2013), while *A. communis* Müll.Arg., a South American species, was cited from India (Rajasekaran and Anandan 2016). Such geographically implausible records strongly suggest misidentification, raising concerns about species attribution and limiting the reliable assignment of findings to specific taxa. While these are clear-cut examples, many other misidentifications are likely more difficult to detect, particularly given the limited taxonomic knowledge available for a large proportion of *Acalypha* species. This pronounced lack of taxonomic verification not only hampers the comparability of studies but also undermines the translational potential of pharmacological research. Addressing this issue is essential for the accurate and reliable documentation of medicinal uses within the genus and for building a trustworthy foundation for future investigations.

### Potential applications and future research directions

Research on *Acalypha* species has revealed promising applications in both medicinal and experimental contexts, yet much remains unexplored. Two areas in particular stand out for their potential: cancer-related activity and the use of *Acalypha* as a pesticide.

### Cancer research and antioxidant potential

Cancer-related studies represent the second most common category of experimental research on *Acalypha*. These works often assess both direct antiproliferative effects and antioxidant properties of plant extracts. Some studies report a reduction in tumour cell mass (Sivakumar et al. 2010), while others highlight the ability of certain extracts to neutralise oxidative stress (Onocha et al. 2011), a factor known to contribute to carcinogenesis (Hanahan and Weinberg 2011; Moloney and Cotter 2018). This distinction is important, as antioxidant activity may have a preventive role even when direct anticancer properties have not been conclusively demonstrated (Raza et al. 2017; Dastmalchi et al. 2020; Luo et al. 2022). Despite these promising findings, the number of *Acalypha* species studied remains small, and experimental designs often lack consistency. More systematic *in vivo* studies are needed to validate these effects and to identify the active compounds responsible.

### Use as pesticide and other applications

Only seven studies mention the use of *Acalypha* species as pesticides targeting insect vectors of human diseases. Three of these (Schmelzer et al. 2008; Quattrocchi 2012; Seebaluck et al. 2015) are secondary sources. Among the remaining four, one provides ethnobotanical information (Kamalakannan and Gopinath 2013), and three present *in vivo* research (Singh et al. 2004; Aboaba et al. 2012; Cruz-Estrada et al. 2013). Five species are documented in this context: *A. alnifolia*, *A. ciliata*, *A. fruticosa*, *A. ornata*, and *A. segetalis*. However, the specific compounds responsible for these effects remain unidentified. Further research is needed to isolate and characterise these bioactive molecules, particularly in light of the growing global demand for environmentally safe alternatives to synthetic pesticides.

### Future research priorities

Several key priorities emerge from our findings:

1. Broaden species coverage. Most experimental studies have focused on a handful of widespread species, while many taxa remain virtually unstudied. Targeted research on these neglected species may reveal novel bioactive properties or unexpected applications.
2. Standardise experimental approaches. The heterogeneity of experimental protocols limits the comparability and reproducibility of results. Adopting common standards for extract preparation, assay selection, and data reporting will allow for more rigorous meta-analyses and more reliable conclusions.
3. Explore non-medicinal uses. Beyond applications in human medicine, *Acalypha* species may hold value in veterinary medicine, pest control, or cultural practices. These potential uses remain largely undocumented and warrant further investigation.

## Conclusion

This review presents the most comprehensive synthesis of medicinal knowledge in *Acalypha* to date, taxonomically validated and systematically structured for subsequent use. By integrating ethnobotanical and experimental data for 62 species across 55 countries, we provide a clear and accessible foundation for future research. Although a few widespread species have received repeated attention, the majority of the genus remains understudied, and research efforts are unevenly distributed across regions. Inconsistencies in taxonomy and methodology limit the usability of published information and obscure broader patterns.

We addressed these challenges through a standardised classification of uses and diseases, rigorous taxonomic verification, and phylogenetic analysis of medicinal taxa. This approach not only improves comparability across studies but also helps identify promising species and neglected research areas. The findings and resources presented here, including a freely available online database, aim to support researchers, practitioners, and conservationists working with medicinal plants.

While this review builds on a strong body of ethnobotanical and pharmacological work, we emphasise the importance of consulting taxonomists and including voucher specimens (deposited in registered herbaria) in future studies to improve traceability, comparability, and reproducibility. Ultimately, unlocking the full potential of *Acalypha* will require closer integration of ethnobotanical knowledge, experimental validation, phytochemical research, and sound taxonomy. We hope this work serves as a starting point for that more collaborative and rigorous path forward.

## Additional information

### Use of AI

No use of AI was reported.

### Author ORCIDs

Lucía Villaescusa-González https://orcid.org/0000-0003-2835-6909

José María Cardiel https://orcid.org/0000-0002-6559-7093

Iris Montero-Muñoz https://orcid.org/0000-0002-8232-0459

Pablo Muñoz-Rodríguez https://orcid.org/0000-0002-3580-8136

### Data availability

All of the data that support the findings of this study are available in the main text or Supplementary Information.

## Supplementary materials

Supplementary material 1 Detailed information on all medicinal uses of *Acalypha* reported in the scientific literature Data type xlsx Explanation note Excel file with detailed, record-by-record information on all medicinal uses of *Acalypha* reported in the scientific literature. https://binary.pensoft.net/file/1523311This dataset is made available under the Open Database License (http://opendatacommons.org/licenses/odbl/1.0/). The Open Database License (ODbL) is a license agreement intended to allow users to freely share, modify, and use this Dataset while maintaining this same freedom for others, provided that the original source and author(s) are credited. Lucía Villaescusa-González, José María Cardiel, Iris Montero-Muñoz, Pablo Muñoz-Rodríguez

Supplementary material 2 Phylogenetic analysis Data type zip https://binary.pensoft.net/file/1523312This dataset is made available under the Open Database License (http://opendatacommons.org/licenses/odbl/1.0/). The Open Database License (ODbL) is a license agreement intended to allow users to freely share, modify, and use this Dataset while maintaining this same freedom for others, provided that the original source and author(s) are credited. Lucía Villaescusa-González, José María Cardiel, Iris Montero-Muñoz, Pablo Muñoz-Rodríguez

Supplementary material 3 Appendices 1, 2 Data type docx Explanation note **appendix 1**. Checklist of ethnomedicinal uses of *Acalypha* species, organised by type of use. **appendix 2**. Checklist of medicinal uses in experimental studies of *Acalypha* species organised by type of study. https://binary.pensoft.net/file/1523313This dataset is made available under the Open Database License (http://opendatacommons.org/licenses/odbl/1.0/). The Open Database License (ODbL) is a license agreement intended to allow users to freely share, modify, and use this Dataset while maintaining this same freedom for others, provided that the original source and author(s) are credited. Lucía Villaescusa-González, José María Cardiel, Iris Montero-Muñoz, Pablo Muñoz-Rodríguez

## References

[B1] Aboaba S, Ibrahim K, Omotoso O (2012) Toxicity and mosquito larvicidal activities of the essential oils from the leaves of *Acalypha ornata* and *Acalypha ciliata* in southwest Nigeria. Journal of Vector Borne Diseases 49: 114–116. 10.4103/0972-9062.213377 22898485

[B2] Alrhmoun M, Sulaiman N, Pieroni A (2024) What drives herbal traditions? The influence of ecology and cultural exchanges on wild plant teas in the Balkan Mountains. Land 13: 2146. 10.3390/land13122146

[B3] Capella-Gutiérrez S, Silla-Martinez JM, Gabaldon T (2009) trimAl: A tool for automated alignment trimming in large-scale phylogenetic analyses. Bioinformatics 25: 1972–1973. 10.1093/bioinformatics/btp348 PMC2712344 19505945

[B4] Cardiel JM, Montero-Muñoz I (2018) Synopsis of *Acalypha* (Euphorbiaceae) of West Tropical Africa, including Cameroon, Chad, Equatorial Guinea, Gabon, and São Tomé and Príncipe. Plant Systematics and Evolution 304: 93–110. 10.1007/s00606-017-1453-4

[B5] Cardiel JM, Muñoz-Rodríguez P (2012) Synopsis of *Acalypha* (Euphorbiaceae) of continental Ecuador. PhytoKeys 17: 1–17. 10.3897/phytokeys.17.3190 PMC3502785 23233813

[B6] Cardiel JM, Rodríguez PM, Muñoz Garmendia F (2013a) Revised taxonomy and nomenclature of *Acalypha* sect. *Communes* (Euphorbiaceae), a complex group of species widespread in the north of the Southern Cone. Taxon 62: 1295–1303. 10.12705/626.11

[B7] Cardiel JM, Nee M, Muñoz Rodríguez P (2013b) Synopsis of *Acalypha* L. (Euphorbiaceae) of Peru and Bolivia, with description of a new species. Anales del Jardín Botánico de Madrid 70: 152–177. 10.3989/ajbm.2366

[B8] Cardiel JM, Muñoz-Rodríguez P, González-Berdasco Á, Montero-Muñoz I (2023) Catalogue and red list of *Acalypha* L. (Euphorbiaceae) from South America. European Journal of Taxonomy 886: 1–92. 10.5852/ejt.2023.886.2201

[B9] Cardiel JM, Muñoz-Rodríguez P, Montero-Muñoz I, Villaescusa-González L, Gamarra R, Ortúñez E (2025) Acalypha Taxonomic Information System (ATIS). https://www.acalypha.es/?AspxAutoDetectCookieSupport=1 [May 5, 2025]

[B10] Cruz-Estrada A, Gamboa-Angulo M, Borges-Argáez R, Ruiz-Sánchez E (2013) Insecticidal effects of plant extracts on immature whitefly *Bemisia tabaci* Genn. (Hemiptera: Aleyroideae). Electronic Journal of Biotechnology 16: 6. 10.2225/vol16-issue1-fulltext-6

[B11] Dastmalchi N, Baradaran B, Latifi-Navid S, Safaralizadeh R, Khojasteh SMB, Amini M, Roshani E, Lotfinejad P (2020) Antioxidants with two faces toward cancer. Life Sciences 258: 118186. 10.1016/j.lfs.2020.118186 32768586

[B12] De Albuquerque UP, Hanazaki N (2009) Five problems in current ethnobotanical research—And some suggestions for strengthening them. Human Ecology 37: 653–661. 10.1007/s10745-009-9259-9

[B13] Hanahan D, Weinberg RA (2011) Hallmarks of cancer: The next generation. Cell 144: 646–674. 10.1016/j.cell.2011.02.013 21376230

[B14] Hart G, Gaoue OG, De La Torre L, Navarrete H, Muriel P, Macía MJ, Balslev H, León-Yánez S, Jørgensen P, Duffy DC (2017) Availability, diversification and versatility explain human selection of introduced plants in Ecuadorian traditional medicine. PLoS ONE 12: e0184369. 10.1371/journal.pone.0184369 PMC5590918 28886104

[B15] Houghton PJ, Howes M-J, Lee CC, Steventon G (2007) Uses and abuses of in vitro tests in ethnopharmacology: Visualizing an elephant. Journal of Ethnopharmacology 110: 391–400. 10.1016/j.jep.2007.01.032 17317057

[B16] Islam MS, Ara H, Ahmad KI, Uddin MM (2019) A review on medicinal uses of different plants of Euphorbiaceae family. Universal Journal of Pharmaceutical Research 4(1): 45–49. 10.22270/ujpr.v4i1.236

[B17] Kalyaanamoorthy S, Minh BQ, Wong TKF, von Haeseler A, Jermiin LS (2017) ModelFinder: Fast model selection for accurate phylogenetic estimates. Nature Methods 14: 587–589. 10.1038/nmeth.4285 PMC5453245 28481363

[B18] Kamalakannan S, Gopinath C (2013) Interaction of *Metarhizium anisopliae* and *Acalypha alnifolia* on the mosquitocidal and IGR activity of Dengue vector, *Aedes aegypti* (L.) (Culicidae: Diptera: Insecta). International Journal of Advanced Biological Research 3: 24–30.

[B19] Katoh K, Standley DM (2013) MAFFT multiple sequence alignment software version 7: Improvements in performance and usability. Molecular Biology and Evolution 30: 772–780. 10.1093/molbev/mst010 PMC3603318 23329690

[B20] Katoh K, Standley DM (2016) A simple method to control over-alignment in the MAFFT multiple sequence alignment program. Bioinformatics 32: 1933–1942. 10.1093/bioinformatics/btw108 PMC4920119 27153688

[B21] Kew RBG (2025) Plants of the World Online | Kew Science. https://powo.science.kew.org/ [May 5, 2025]

[B22] Levin GA, Cardinal-McTeague WM, Steinmann VW, Sagun VG (2022) Phylogeny, classification, and character evolution of *Acalypha* (Euphorbiaceae: Acalyphoideae). Systematic Botany 47: 477–497. 10.1600/036364422X16512572275034

[B23] Luo M, Zhou L, Huang Z, Li B, Nice EC, Xu J, Huang C (2022) Antioxidant therapy in cancer: rationale and progress. Antioxidants 11: 1128. 10.3390/antiox11061128 PMC9220137 35740025

[B24] Malaspina P, Betuzzi F, Ingegneri M, Smeriglio A, Cornara L, Trombetta D (2022) Risk of poisoning from garden plants: Misidentification between laurel and cherry laurel. Toxins 14: 726. 10.3390/toxins14110726 PMC9697506 36355976

[B25] Mateo‐Martín J, Benítez G, Gras A, Molina M, Reyes‐García V, Tardío J, Verde A, Pardo‐de‐Santayana M (2023) Cultural importance, availability and conservation status of Spanish wild medicinal plants: Implications for sustainability. People and Nature 5: 1512–1525. 10.1002/pan3.10511

[B26] Minh BQ, Schmidt HA, Chernomor O, Schrempf D, Woodhams MD, Von Haeseler A, Lanfear R (2020) IQ-TREE 2: new models and efficient methods for phylogenetic inference in the genomic era. Molecular Biology and Evolution 37: 1530–1534. 10.1093/molbev/msaa015 PMC7182206 32011700

[B27] Moloney JN, Cotter TG (2018) ROS signalling in the biology of cancer. Seminars in Cell & Developmental Biology 80: 50–64. 10.1016/j.semcdb.2017.05.023 28587975

[B28] Montero-Muñoz I, Levin GA, Cardiel JM (2023) Monograph of *Acalypha* L. (Euphorbiaceae) of the Western Indian Ocean Region, with the description of a new species from Mayotte. Adansonia 45(26): 395–496. 10.5252/adansonia2023v45a26

[B29] Mwine JT, Van Damme P (2011) Why do Euphorbiaceae tick as medicinal Plants? A review of Euphorbiaceae family and its medicinal features. Journal of Medicinal Plants Research 5: 652–662.

[B30] Noumedem JA, Tamokou J de D, Teke GN, Momo RC, Kuete V, Kuiate JR (2013) Phytochemical analysis, antimicrobial and radical-scavenging properties of Acalyphamanniana leaves. SpringerPlus 2: 503. 10.1186/2193-1801-2-503 PMC3795206 24130962

[B31] Onocha P, Oloyede G, Afolabi OO (2011) Chemical composition, cytotoxicity and antioxidant activity of essential oils of *Acalypha hispida* flowers. International Journal of Pharmacology 7(1): 144–148. 10.3923/ijp.2011.144.148

[B32] Pardo de Santayana M, Morales R, Aceituno L, Molina M (2014) Inventario español de los conocimientos tradicionales relativos a la biodiversidad. Ministerio de Agricultura, Alimentación y Medio Ambiente, Madrid, 411 pp.

[B33] Parnmen S, Nooron N, Sikaphan S, Pringsulaka O, Rangsiruji A (2023) Potential toxicity of wild *Ipomoea* ingested by schoolchildren in remote Northeastern Thailand. Journal of Associated Medical Sciences 56: 54–62. 10.12982/JAMS.2023.008

[B34] Quattrocchi U (2012) CRC World dictionary of medicinal and poisonous plants: common names, scientific names, eponyms, synonyms, and etymology (5 Vol. Set). CRC Press, 4038 pp.

[B35] Rajasekaran S, Anandan R (2016) Phytochemical and pharmacological evaluation of *Acalypha communis* Müll.Arg. for their hepatoprotective activity. Asian Journal of Pharmaceutical and Clinical Research, 94–97.

[B36] Raza MH, Siraj S, Arshad A, Waheed U, Aldakheel F, Alduraywish S, Arshad M (2017) ROS-modulated therapeutic approaches in cancer treatment. Journal of Cancer Research and Clinical Oncology 143: 1789–1809. 10.1007/s00432-017-2464-9 PMC11819417 28647857

[B37] Sagun VG, Levin GA, van Welzen PC (2010) Revision and phylogeny of *Acalypha* (Euphorbiaceae) in malesia. Blumea 55: 21–60. 10.3767/000651910X499141

[B38] Schmelzer GH, Gurib-Fakim A, Schmelzer GH, Foundation P (2008) Medicinal Plants. PROTA, 793 pp.

[B39] Seebaluck R, Gurib-Fakim A, Mahomoodally F (2015) Medicinal plants from the genus *Acalypha* (Euphorbiaceae). A review of their ethnopharmacology and phytochemistry. Journal of Ethnopharmacology 159: 137–157. 10.1016/j.jep.2014.10.040 25446604

[B40] Singh DAP, Raman M, Saradha V, Jayabharathi P, Kumar VRS (2004) Acaricidal property of kuppaimeni (*Acalypha indica*) against natural *Psoroptes cuniculi* infestation in broiler rabbits. The Indian Journal of Animal Sciences 74(10). https://epubs.icar.org.in/index.php/IJAnS/article/view/39275 [January 2, 2024]

[B41] Sivakumar T, Murthi MNV, Kumutha P (2010) Evaluation of anti-tumor and anti-oxidant activity of *Acalypha fruticosa* in Ehrlich’s Ascites Carcinoma bearing Swiss albino mice. Research Journal of Pharmaceutical, Biological and Chemical Sciences 1: 191–199.

[B42] Soladoye M, Sonibare M, Rosanwo T (2008) Phytochemical and morphometric analysis of the genus *Acalypha* Linn. (Euphorbiaceae). Journal of Applied Sciences 8(17): 3044–3049. 10.3923/jas.2008.3044.3049

[B43] Staub PO, Geck MS, Weckerle CS, Casu L, Leonti M (2015) Classifying diseases and remedies in ethnomedicine and ethnopharmacology. Journal of Ethnopharmacology 174: 514–519. 10.1016/j.jep.2015.08.051 26342522

[B44] Wheeler QD (2024) Species, science and society: the role of systematic biology. Routledge, New York, NY. 10.4324/9781003389071

[B45] World Health Organization (2022) International Classification of Diseases for Mortality and Morbidity Statistics. 11^th^ Version. World Health Organization, Geneva. https://icd.who.int/browse11/l-m/en [January 11, 2024]

[B46] Zahidin NS, Saidin S, Zulkifli RM, Muhamad II, Ya’akob H, Nur H (2017) A review of *Acalypha indica* L. (Euphorbiaceae) as traditional medicinal plant and its therapeutic potential. Journal of Ethnopharmacology 207: 146–173. 10.1016/j.jep.2017.06.019 28647509

