# Supplementary figures and images for: Revisiting Acalypha medicinal interest: ethnobotany, experimental studies, and the implications of taxonomic misuse pitfalls
Source: PhytoKeys. 2026 Jan 30;270:119–42. doi: 10.3897/phytokeys.270.169087 (PMC12881909; doi:10.3897/phytokeys.270.169087)

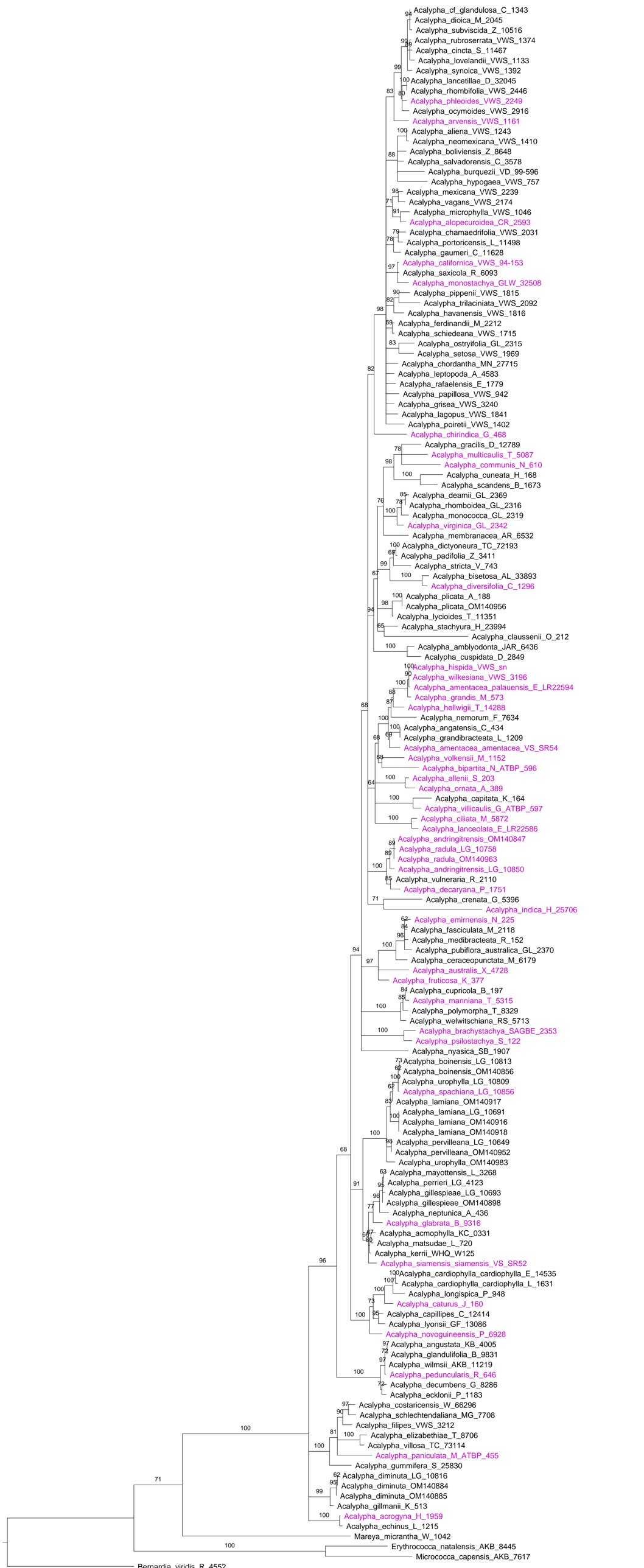

Supplement: Supplementary material 2 — Phylogenetic analysis [file phytokeys-270-119_article-169087__-s002.zip › phylogenetic_analysis/phylogeny_with_names.pdf]
