# Supplementary material for: Revisiting Acalypha medicinal interest: ethnobotany, experimental studies, and the implications of taxonomic misuse pitfalls
Source: PhytoKeys. 2026 Jan 30;270:119–42. doi: 10.3897/phytokeys.270.169087 (PMC12881909; doi:10.3897/phytokeys.270.169087)
Supplement: Supplementary material 3 — Appendices 1, 2 [file phytokeys-270-119_article-169087__-s003.docx]

**APPENDICES**

## *Appendix 1.* Checklist of ethnomedicinal uses of *Acalypha* species, organised by type of use. Asterisks (*) indicate secondary sources (i.e., a compilation rather than original research).

1. Human medicine

| **Type of disease** | | **Species** | **References** |
| --- | --- | --- | --- |
| **First classification** | **Second classification** |  |  |
| **Cardiovascular system** | - | *Acalypha alnifolia* J.G. Klein ex Willd. | (Johnkennedy et al., 2011)  (Seebaluck et al., 2015)* |
|  |  | *Acalypha indica* L. | (Quattrocchi, 2012)*  (Seebaluck et al., 2015)* |
|  |  | *Acalypha multicaulis* Müll.Arg. | (de Albuquerque et al., 2007)* |
|  |  | *Acalypha reflexa* Müll.Arg. | (Bussmann et al., 2010)*  (Seebaluck et al., 2015)* |
|  |  | *Acalypha wilkesiana* Müll.Arg. | (Adesina et al., 2000)  (Akinyemi et al., 2005)  (Gbolade, 2012)  (C. J. Ikewuchi et al., 2009; Ikewuchi et al., 2010, 2011; J. C. Ikewuchi et al., 2009)  (Mendame et al., 2022)  (Oliver-Bever, 1986)  (Quds et al., 2012)  (Seebaluck et al., 2015)* |
| **Digestive tract** | - | *Acalypha alopecuroidea* Jacq. | (Argueta et al., 1994)*  (Beyra et al., 2004)*  (Carmona et al., 2008)  (Johnson, 2019)*  (Madlener et al., 2009)  (Quattrocchi, 2012)*  (Seebaluck et al., 2015)*  (Svačinová, 2011)* |
|  |  | *Acalypha ambigua* Pax | (Quattrocchi, 2012)* |
|  |  | *Acalypha arvensis* Poepp. & Endl. | (Arvigo and Balick, 1993)*  (House et al., 1989) |
|  |  | *Acalypha califórnica* Benth. | (Alonso-Castro et al., 2011)* |
|  |  | *Acalypha ciliata* Forssk. | (Adsul et al., 2013)  (Quattrocchi, 2012)* |
|  |  | *Acalypha communis* Müll.Arg. | (Arias, 2009) |
|  |  | *Acalypha decaryana* Leandri | (Quattrocchi, 2012)*  (Seebaluck et al., 2015)* |
|  |  | *Acalypha fruticosa* Forrsk. | (Bama et al., 2013)  (Bijekar and Gayatri, 2014)*  (Drury, 1873)*  (Ichikawa, 1987)  (Kirtikar and Basu, 1935)*  (Quattrocchi, 2012)*  (Seebaluck et al., 2015)*  (Schmelzer et al., 2008)*  (Todd., 1888) |
|  |  | *Acalypha hispida* Burm. f. | (Adsul et al., 2013)  (Bokshi et al., 2012)  (P. Onocha et al., 2011a, 2011b; P. A. Onocha et al., 2011)  (Quattrocchi, 2012)*  (Seebaluck et al., 2015)* |
|  |  | *Acalypha indica* L. | (Aishwarya et al., 2024)*  (Das et al., 2005)  (Dineshkumar et al., 2010)*  (Drury, 1873)*  (Dymock, 1884)*  (Eswaran et al., 2013)  (Ganesan and Xu, 2017)  (Islam et al., 2019)*  (Kirtikar, 1935)*  (Pragada et al., 2011)*  (Quattrocchi, 2012)*  (Seebaluck et al., 2015)*  (Schmelzer et al., 2008)*  (Senthilkumar et al., 2006)  (Sudheer et al., 2019)  (van Valkenburg and Bunyapraphatsara, 2001)*  (Venkateshwarlu and Sridhar, 2017)  (Zahidin et al., 2017)* |
|  |  | *Acalypha integrifolia* Willd. | (Quattrocchi, 2012)*  (Seebaluck et al., 2015)* |
|  |  | *Acalypha lanceolata* Willd. | (van Valkenburg and Bunyapraphatsara, 2001)*  (Quattrocchi, 2012)* |
|  |  | *Acalypha ornata* Hochst. ex. A.Rich. | (Aboaba et al., 2012)*  (Burkill, 1994)*  (Quattrocchi, 2012)*  (Schmelzer et al., 2008)* |
|  |  | *Acalypha paniculata* Miq. | (Quattrocchi, 2012)* |
|  |  | *Acalypha siamensis* Oliv. ex. Gage | (van Valkenburg and Bunyapraphatsara, 2001)*  (Ng and Songkhla, 2000)  (Quattrocchi, 2012)*  (Seebaluck et al., 2015)*  (van Valkenburg and Bunyapraphatsara, 2001)*  (Wiart et al., 2004) |
|  |  | *Acalypha villicaulis* Hochst. ex. A.Rich. | (Schmelzer et al., 2008)*  (Ramathal and Ngassapa, 2001) |
|  |  | *Acalypha volkensii* Pax | (Quattrocchi, 2012)* |
|  |  | *Acalypha wilkesiana* Müll.Arg. | (Adsul et al., 2013)  (Akinyemi et al., 2005)  (J. C. Ikewuchi et al., 2009)  (Odugbemi, 2008)*  (Quattrocchi, 2012)*  (Quds et al., 2012)  (Seebaluck et al., 2015)*  (van Valkenburg and Bunyapraphatsara, 2001)*  (World Health Organization (WHO), 2009)* |
| **Urinary system** | - | *Acalypha alopecuroidea* Jacq. | (Carmona et al., 2008)  (Madlener et al., 2009)  (Quattrocchi, 2012)*  (Svačinová, 2011) |
|  |  | *Acalypha arvensis* Poepp. | (Arvigo and Balick, 1993)* |
|  |  | *Acalypha hispida* Burm. f. | (Bokshi et al., 2012)  (P. Onocha et al., 2011a, 2011b; P. A. Onocha et al., 2011)  (Quattrocchi, 2012)*  (Seebaluck et al., 2015)* |
|  |  | *Acalypha indica* L. | (Das et al., 2005)  (Ganesan and Xu, 2017)  (Kirtikar, 1935)*  (Quattrocchi, 2012)*  (Seebaluck et al., 2015)*  (Senthilkumar et al., 2006)* |
|  |  | *Acalypha siamensis* Oliv. ex. Gage | (Ng and Songkhla, 2000)  (Quattrocchi, 2012)*  (Seebaluck et al., 2015)*  (van Valkenburg and Bunyapraphatsara, 2001)*  (Wiart et al., 2004) |
|  |  | *Acalypha villicaulis* Hochst. ex. A.Rich. | (Seebaluck et al., 2015)*  (Ramathal and Ngassapa, 2001) |
|  |  | *Acalypha virginica* L. | (Hyams, 1898)*  (Raleigh, 1898)* |
|  |  | *Acalypha volkensii* Pax | (Quattrocchi, 2012)* |
|  |  | *Acalypha wilkesiana* Müll.Arg. | (Adesina et al., 2000)  (C. J. Ikewuchi et al., 2009; Ikewuchi et al., 2010, 2011; J. C. Ikewuchi et al., 2009)  (Oliver-Bever, 1986)  (Seebaluck et al., 2015)* |
| **Pregnancy, childbirth and puerperium** | - | *Acalypha adenostachya* Müll.Arg. | (Hurtado Rico et al., 2006) |
|  |  | *Acalypha fruticosa* Forssk. | (Pakia et al., 2003) |
|  |  | *Acalypha monostachya* Cav. | (Canales et al., 2005) |
|  |  | *Acalypha ornata* Hochst. Ex. A.Rich. | (Aboaba et al., 2012)  (Burkill, 1994)*  (Quattrocchi, 2012)*  (Sabrina et al., 2005)  (Schmelzer et al., 2008)* |
|  |  | *Acalypha phleoides* Cav. | (Hurtado Rico et al., 2006) |
|  |  | *Acalypha psilostachya* Hochst. Ex. A.Rich. | (Neuwinger, 2000)*  (Quattrocchi, 2012)*  (Schmelzer et al., 2008)* |
| **Perinatal period** | - | *Acalypha ornata* Hochst. Ex A.Rich. | (Schmelzer et al., 2008)* |
| **Reproductive system** | Fertility | *Acalypha ciliata* Forssk. | (Aboaba et al., 2012)  (Quattrocchi, 2012)*  (Schmelzer et al., 2008)*  (Seebaluck et al., 2015)* |
|  |  | *Acalypha villicaulis* Hochst. Ex. A.Rich. | (Quattrocchi, 2012)*  (Schmelzer et al., 2008)*  (Seebaluck et al., 2015)* |
|  | Abortifacient / Contraceptive | *Acalypha villicaulis* Hochst. Ex. A.Rich. | (Schmelzer et al., 2008)*  (Seebaluck et al., 2015)* |
|  |  | *Acalypha wilkesiana* Müll.Arg. | (Kumar et al., 2012)*  (Seebaluck et al., 2015)*  (World Health Organization (WHO), 2009)* |
|  | Others | *Acalypha allenii* Hutch. | (Quattrocchi, 2012)* |
|  |  | *Acalypha fruticosa* Forssk. | (Quattrocchi, 2012)*  (Schmelzer et al., 2008)* |
|  |  | *Acalypha indica* L. | (Quattrocchi, 2012)*  (Zahidin et al., 2017)* |
|  |  | *Acalypha ornata* Hochst. Ex. A.Rich. | (Aboaba et al., 2012)  (Burkill, 1994)*  (Quattrocchi, 2012)*  (Schmelzer et al., 2008)* |
|  |  | *Acalypha villicaulis* Hochst. Ex. A.Rich. | (Quattrocchi, 2012)* |
| **Endocrine – metabolic system** | - | *Acalypha alnifolia* J.G. Klein ex Willd. | (Balakrishnan et al., 2009)  (Kovendan et al., 2013)  (Revathi et al., 2013)  (Seebaluck et al., 2015)* |
|  |  | *Acalypha indica* L. | (Ganesan and Xu, 2017)  (Zahidin et al., 2017)* |
|  |  | *Acalypha wilkesiana* Müll.Arg. | (Adesina et al., 2000)  (Akinyemi et al., 2005)  (C. J. Ikewuchi et al., 2009; Ikewuchi et al., 2010, 2011; J. C. Ikewuchi et al., 2009)  (Mendame et al., 2022)  (Oliver-Bever, 1986)  (Quds et al., 2012)  (Seebaluck et al., 2015)* |
| **Respiratory system** | - | *Acalypha alopecuroidea* Jacq. | (Alonso-Castro et al., 2011)*  (Argueta et al., 1994)*  (Del Rosario Pérez and Weniger, 1988)  (Hernández et al., 2003)  (Johnson, 2019)*  (Madlener et al., 2009)  (Seebaluck et al., 2015)*  (Svačinová, 2011) |
|  |  | *Acalypha australis* L. | (Dong et al., 1994)  (Quattrocchi, 2012)*  (Seebaluck et al., 2015)*  (van Valkenburg and Bunyapraphatsara, 2001)* |
|  |  | *Acalypha ciliata* Forssk. | (Odugbemi, 2008)*  (Quattrocchi, 2012)*  (Quds et al., 2012)  (Seebaluck et al., 2015)* |
|  |  | *Acalypha fruticosa* Forssk. | (Hassan-Abdallah et al., 2013)  (Khan, 2001)  (Quattrocchi, 2012)*  (Ruffo et al., 2002)*  (Schmelzer et al., 2008)*  (Seebaluck et al., 2015)* |
|  |  | *Acalypha grandis* Benth. | (Bradacs et al., 2010) |
|  |  | *Acalypha hispida* Burm. f. | (Adsul et al., 2013)  (Bokshi et al., 2012)  (P. Onocha et al., 2011a; P. A. Onocha et al., 2011; Quds et al., 2012)  (Quattrocchi, 2012)*  (Seebaluck et al., 2015)*  (van Valkenburg and Bunyapraphatsara, 2001)* |
|  |  | *Acalypha indica* L. | (Kirtikar and Basu, 1935)*  (Das et al., 2005)  (Dineshkumar et al., 2010)  (Drury, 1873)*  (Dymock, 1884)*  (Ganesan and Xu, 2017)  (Islam et al., 2019)*  (Odugbemi, 2008)  (Pandit, 2010)  (Quattrocchi, 2012)* (Rao et al., 2008)  (Schmelzer et al., 2008)*  (Seebaluck et al., 2015)*  (Senthilkumar et al., 2006)  (van Valkenburg and Bunyapraphatsara, 2001)*  (Zahidin et al., 2017)* |
|  |  | *Acalypha paniculata* Miq. | (Quattrocchi, 2012)* |
|  |  | *Acalypha peduncularis* Meisn. Ex. C.Krauss | (Quattrocchi, 2012)* |
|  |  | *Acalypha siamensis* Oliv. Ex. Gage | (van Valkenburg and Bunyapraphatsara, 2001)* |
|  |  | *Acalypha villicaulis* Hochst. Ex. A.Rich. | (Quattrocchi, 2012)*  (Schmelzer et al., 2008)* |
|  |  | *Acalypha virginica* L. | (Hyams, 1898)*  (Porcher, 1849)*  (Raleigh, 1898)*  (Williams, 1849)* |
|  |  | *Acalypha volkensii* Pax | (Quattrocchi, 2012)* |
|  |  | *Acalypha wilkesiana* Müll.Arg. | (Odugbemi, 2008)*  (Quattrocchi, 2012)*  (Seebaluck et al., 2015)*  (van Valkenburg and Bunyapraphatsara, 2001)*  (World Health Organization  (WHO), 2009)* |
| **Immune system** | - | *Acalypha wilkesiana* Müll.Arg. | (Odugbemi, 2008)* |
| **Locomotor system** | - | *Acalypha ciliata* Forssk. | (Odugbemi, 2008)*  (Quds et al., 2012)  (Seebaluck et al., 2015)* |
|  |  | *Acalypha emirnensis* Baill. | (Quattrocchi, 2012)*  (Schmelzer et al., 2008)*  (Seebaluck et al., 2015)* |
|  |  | *Acalypha fruticosa* Forssk. | (Bama et al., 2013)  (Seebaluck et al., 2015)* |
|  |  | *Acalypha idica* L. | (Aishwarya et al., 2024)*  (Cooper, 1842)*  (Dineshkumar et al., 2010)*  (Drury, 1873)*  (Islam et al., 2019)*  (Kirtikar and Basu, 1935)*  (Odugbemi, 2008)*  (Quattrocchi, 2012)*  (Schmelzer et al., 2008)*  (van Valkenburg and Bunyapraphatsara, 2001)*  (Venkateshwarlu and Sridhar, 2017) |
|  |  | *Acalypha paniculata* Miq. | (Ramya, 2008) |
|  |  | *Acalypha wilkesiana* Müll.Arg. | (van Valkenburg and Bunyapraphatsara, 2001)* |
| **Skin** | - | *Acalypha alopecuroidea* Jacq. | (Madlener et al., 2009)  (Quattrocchi, 2012)*  (Seebaluck et al., 2015)*  (Svačinová, 2011) |
|  |  | *Acalypha andringitrensis* Leandri | (Quattrocchi, 2012)*  (Schmelzer et al., 2008)*  (Seebaluck et al., 2015)* |
|  |  | *Acalypha arvensis* Poepp. | (Arvigo and Balick, 1993)*  (House et al., 1989) |
|  |  | *Acalypha australis* L. | (Dong et al., 1994)  (Quattrocchi, 2012)*  (Seebaluck et al., 2015)* |
|  |  | *Acalypha caturus* Blume | (van Valkenburg and Bunyapraphatsara, 2001)* |
|  |  | *Acalypha ciliata* Forssk. | (Odugbemi, 2008)* |
|  |  | *Acalypha fruticosa* Forssk. | (Bama et al., 2013)  (Bijekar and Gayatri, 2014)*  (Hassan-Abdallah et al., 2013)  (Hedberg et al., 1982)  (Ignacimuthu et al., 2006)  (Mohagheghzadeh et al., 2006)*  (Quattrocchi, 2012)*  (Schmelzer et al., 2008)* (Seebaluck et al., 2015)* |
|  |  | *Acalypha glabrata* Thunb. | (Afolayan et al., 2014)  (Bhat, 2013)  (Dlisani and Bhat, 1999)  (Ndhlovu et al., 2021)* |
|  |  | *Acalypha grandis* Benth. | (van Valkenburg and Bunyapraphatsara, 2001)* |
|  |  | *Acalypha hellwigii* Warb. | (van Valkenburg and Bunyapraphatsara, 2001)* |
|  |  | *Acalypha hispida* Burm. f. | (Adsul et al., 2013)  (Quattrocchi, 2012)* |
|  |  | *Acalypha indica* L. | (Aishwarya et al., 2024)*  (Das et al., 2005)  (Dineshkumar et al., 2010)*  (Drury, 1873)*  (Ganesan and Xu, 2017)  (Islam et al., 2019)*  (Kirtikar, 1935)*  (Kiruba et al., 2006)  (Kumar et al., 2012)  (Mallik et al., 2012)  (Quattrocchi, 2012)*  (Schmelzer et al., 2008)*  (Seebaluck et al., 2015)*  (Todd., 1888)  (van Valkenburg and Bunyapraphatsara, 2001)*  (Venkateshwarlu and Sridhar, 2017)  (Zahidin et al., 2017)* |
|  |  | *Acalypha insulana* Müll.Arg. | (Quattrocchi, 2012)*  (Worth and Sakulas, 1987) |
|  |  | *Acalypha integrifolia* Willd. | (Quattrocchi, 2012)*  (Schmelzer et al., 2008)* |
|  |  | *Acalypha lanceolata* Willd. | (Quattrocchi, 2012)* |
|  |  | *Acalypha ornata* Hochst. Ex. A.Rich. | (Aboaba et al., 2012)  (Burkill, 1994)*  (Odugbemi, 2008)*  (Schmelzer et al., 2008)* |
|  |  | *Acalypha paniculata* Miq. | (Ignacimuthu et al., 2006)  (Quattrocchi, 2012)* |
|  |  | *Acalypha rádula* Baker | (Quattrocchi, 2012)* |
|  |  | *Acalypha villicaulis* Hochst. Ex. A. Rich | (Schmelzer et al., 2008)* |
|  |  | *Acalypha volkensii* Pax | (Quattrocchi, 2012)* |
|  |  | *Acalypha wilkesiana* Müll.Arg. | (Adsul et al., 2013)  (Akinyemi et al., 2005)  (Odugbemi, 2008)*  (Quds et al., 2012)  (Seebaluck et al., 2015)* |
| **Nervous system** | - | *Acalypha fruticosa* Forssk. | (Kirtikar, 1935)  (Quattrocchi, 2012)*  (Schmelzer et al., 2008)* |
|  |  | *Acalypha indica* L. | (Quattrocchi, 2012)*  (Zahidin et al., 2017)* |
|  |  | *Acalypha ornata* Hochst. Ex A.Rich. | (Mohagheghzadeh et al., 2006)  (Moshi et al., 2005) |
| **Eye problems** | - | *Acalypha fruticosa* Forssk. | (Quattrocchi, 2012)*  (Ruffo et al., 2002)*  (Schmelzer et al., 2008)*  (Seebaluck et al., 2015)* |
|  |  | *Acalypha indica* L. | (Quattrocchi, 2012)*  (Schmelzer et al., 2008)* |
|  |  | *Acalypha novoguineensis* Warb. | (Quattrocchi, 2012)*  (Worth and Sakulas, 1987) |
|  |  | *Acalypha psilostachya* Hochst. Ex A.Rich. | (Neuwinger, 2000)(Schmelzer et al., 2008)  (Quattrocchi, 2012)*  (Schmelzer et al., 2008)* |
| **Ear problems** | - | *Acalypha indica* L. | (Drury, 1873)*  (Dymock, 1884)*  (Quattrocchi, 2012)* |
| **Mouth problems** | - | *Acalypha fruticosa* Forssk. | (Hassan-Abdallah et al., 2013)  (Quattrocchi, 2012)*  (Ruffo et al., 2002)*  (Schmelzer et al., 2008)*  (Seebaluck et al., 2015)* |
|  |  | *Acalypha indica* L. | (Zahidin et al., 2017)* |
|  |  | *Acalypha multicaulis* Müll.Arg. | (de Albuquerque et al., 2007)* |
| **STD** | - | *Acalypha andringitrensis* Leandri | (Quattrocchi, 2012)*  (Schmelzer et al., 2008)*  (Seebaluck et al., 2015)* |
|  |  | *Acalypha ciliata* Forskk. | (Odugbemi, 2008)*  (Quds et al., 2012)  (Seebaluck et al., 2015)* |
|  |  | *Acalypha fruticosa* Forssk. | (Quattrocchi, 2012)*  (Ruffo et al., 2002)*  (Schmelzer et al., 2008)*  (Seebaluck et al., 2015)* |
|  |  | *Acalypha hispida* Burm. F. | (Quattrocchi, 2012)*  (van Valkenburg and Bunyapraphatsara, 2001)* |
|  |  | *Acalypha indica* L. | (Dineshkumar et al., 2010)*  (Seebaluck et al., 2015)*  (Senthilkumar et al., 2006)*  (Zahidin et al., 2017)* |
|  |  | *Acalypha radula* Baker | (Quattrocchi, 2012)* |
|  |  | *Acalypha spachiana* Baill. | (Quattrocchi, 2012)* |
|  |  | *Acalypha volkensii* Pax | (Quattrocchi, 2012)* |
| **Other infectious or parasitic diseases** | - | *Acalypha alnifolia* J.G. Klein ex Willd. | (Quattrocchi, 2012)*  (Rao et al., 2008)  (Revathi et al., 2013)  (Seebaluck et al., 2015)* |
|  |  | *Acalypha alopecuroidea* Jacq. | (Argueta et al., 1994)*  (Johnson, 2019)*  (Madlener et al., 2009)  (Seebaluck et al., 2015)*  (Svačinová, 2011) |
|  |  | *Acalypha amentacea* Roxb. | (Quattrocchi, 2012)* |
|  |  | *Acalypha andringitrensis* Leandri | (Quattrocchi, 2012)*  (Schmelzer et al., 2008)  (Seebaluck et al., 2015)* |
|  |  | *Acalypha arvensis* Poepp. | (Arvigo and Balick, 1993)* |
|  |  | *Acalypha australis* L. | (Dong et al., 1994)  (Quattrocchi, 2012)*  (Seebaluck et al., 2015)*  (van Valkenburg and Bunyapraphatsara, 2001)* |
|  |  | *Acalypha ciliata* Forssk. | (Adsul et al., 2013)  (Odugbemi, 2008)*  (Quattrocchi, 2012)*  (Schmelzer et al., 2008)* |
|  |  | *Acalypha decaryana* Leandri | (Quattrocchi, 2012)*  (Seebaluck et al., 2015)* |
|  |  | *Acalypha filiformis* Poir. | (Seebaluck et al., 2015)* |
|  |  | *Acalypha fruticosa* Forssk. | (Bama et al., 2013)  (Drury, 1873)*  (Hassan-Abdallah et al., 2013)  (Ignacimuthu et al., 2006)  (Khan, 2001)  (Quattrocchi, 2012)*  (Ruffo et al., 2002)*  (Seebaluck et al., 2015)*  (Todd., 1888) |
|  |  | *Acalypha grandis* Benth. | (McClatchey, 1996)  (Seebaluck et al., 2015)*  (van Valkenburg and Bunyapraphatsara, 2001)*  (World Health Organization (WHO), 2009)* |
|  |  | *Acalypha hispida* Burm. F. | (Adsul et al., 2013)  (Bokshi et al., 2012)  (McLaughlin, 1994)  (P. Onocha et al., 2011a, 2011b; P. A. Onocha et al., 2011)  (Quattrocchi, 2012)*  (van Valkenburg and Bunyapraphatsara, 2001)* |
|  |  | *Acalypha indica* L. | (Aishwarya et al., 2024)*  (Bandyopadhyay and Mukherjee, 2005)  (Dineshkumar et al., 2010)*  (Dymock, 1884)*  (Eswaran et al., 2013)  (Ganesan and Xu, 2017)  (Giday and Teklehaymanot, 2013)  (Islam et al., 2019)*  (Kirtikar, 1935)  (Lakshminarayana and Narasimharao, 2013)  (Noriko, 2013)  (Pandit, 2010)  (Quattrocchi, 2012)*  (Rao et al., 2008)  (Schmelzer et al., 2008)*  (Seebaluck et al., 2015)*  (Todd., 1888)  (van Valkenburg and Bunyapraphatsara, 2001)*  (Zahidin et al., 2017)* |
|  |  | *Acalypha integrifolia* Willd. | (Quattrocchi, 2012)*  (Schmelzer et al., 2008)* |
|  |  | *Acalypha ornata* Hochst. Ex A.Rich. | (Aboaba et al., 2012)  (Burkill, 1994)*  (Quattrocchi, 2012)*  (Ruffo et al., 2002)*  (Schmelzer et al., 2008)* |
|  |  | *Acalypha paniculata* Miq. | (Quattrocchi, 2012)* |
|  |  | *Acalypha psilostachya* Hochst. Ex A.Rich. | (Neuwinger, 2000)  (Quattrocchi, 2012)*  (Schmelzer et al., 2008)* |
|  |  | *Acalypha radula* Baker | (Quattrocchi, 2012)* |
|  |  | *Acalypha siamensis* Oliv. Ex Gage | (van Valkenburg and Bunyapraphatsara, 2001)* |
|  |  | *Acalypha spachiana* Bail. | (Quattrocchi, 2012)* |
|  |  | *Acalypha villicaulis* Hochst. Ex A.Rich. | (Quattrocchi, 2012)* |
|  |  | *Acalypha volkensii* Pax | (Quattrocchi, 2012)* |
|  |  | *Acalypha wilkesiana* Müll.Arg. | (Adesina et al., 2000)  (Akinyemi et al., 2005)  (C. J. Ikewuchi et al., 2009; Ikewuchi et al., 2010, 2011; J. C. Ikewuchi et al., 2009)  (Odugbemi, 2008)*  (Oliver-Bever, 1986)  (Quattrocchi, 2012)*  (Quds et al., 2012)  (van Valkenburg and Bunyapraphatsara, 2001)*  (World Health Organization (WHO), 2009)* |
| **Neoplasm** | - | *Acalypha acrogyna* Pax | (Ayele, 2018)  (Ragunathan and Solomon, 2012) |
|  |  | *Acalypha alopecuroidea* Jacq. | (Argueta et al., 1994)*  (Carmona et al., 2008)  (Johnson, 2019)*  (Madlener et al., 2009)  (Quattrocchi, 2012)*  (Svačinová, 2011) |
|  |  | *Acalypha californica* Benth. | (Moreno-Salazar et al., 2008)  (Robles-Zepeda et al., 2013) |
|  |  | *Acalypha fruticosa* Forssk. | (Bijekar and Gayatri, 2014)* |
|  |  | *Acalypha grandis* Benth. | (van Valkenburg and Bunyapraphatsara, 2001) |
|  |  | *Acalypha wilkesiana* Müll.Arg. | (Büssing et al., 1999)  (Quds et al., 2012)  (Seebaluck et al., 2015)* |
| **Symptoms** | Pain-killer | *Acalypha brachystahcya* Hornem. | (Quattrocchi, 2012)* |
|  |  | *Acalypha caturus* Blume | (van Valkenburg and Bunyapraphatsara, 2001)* |
|  |  | *Acalypha chirindica* S.Moore | (Gordon, 2017) |
|  |  | *Acalypha ciliata* Forssk. | (Quattrocchi, 2012)* |
|  |  | *Acalypha engleri* Pax | (Quattrocchi, 2012)* |
|  |  | *Acalypha fruticosa* Forssk. | (Khan, 2001)  (Quattrocchi, 2012)*  (Ruffo et al., 2002)*  (Schmelzer et al., 2008)* |
|  |  | *Acalypha indica* L. | (Aishwarya et al., 2024)*  (Das et al., 2005)  (Dineshkumar et al., 2010)*  (Ganesan and Xu, 2017)  (Kirtikar and Basu, 1935)  (Quattrocchi, 2012)*  (Schmelzer et al., 2008)*  (Venkateshwarlu and Sridhar, 2017)  (Zahidin et al., 2017)* |
|  |  | *Acalypha lanceolata* Willd. | (Quattrocchi, 2012)* |
|  |  | *Acalypha ornata* Hochst. Ex A.Rich. | (Quattrocchi, 2012)*  (Schmelzer et al., 2008)* |
|  |  | *Acalypha paniculata* Miq. | (Ignacimuthu et al., 2006)  (Quattrocchi, 2012)* |
|  |  | *Acalypha psilostachya* Hochst. Ex A.Rich. | (Neuwinger, 2000)  (Quattrocchi, 2012)*  (Schmelzer et al., 2008)* |
|  |  | *Acalypha wilkesiana* Müll.Arg. | (Odugbemi, 2008)*  (Mendame et al., 2022)  (Quattrocchi, 2012)* |
|  | Diarrhea | *Acalypha allenii* Hutch. | (Quattrocchi, 2012)* |
|  |  | *Acalypha alopecuroidea*  Jacq. | (Argueta et al., 1994)*  (Del Rosario Pérez and Weniger, 1988)  (Johnson, 2019)*  (Madlener et al., 2009)  (Seebaluck et al., 2015)*  (Svačinová, 2011) |
|  |  | *Acalypha amentacea* Roxb. | (Quattrocchi, 2012)* |
|  |  | *Acalypha australis* L. | (Das et al., 2005)  (Dong et al., 1994)  (Quattrocchi, 2012)*  (Seebaluck et al., 2015)  (van Valkenburg and Bunyapraphatsara, 2001)* |
|  |  | *Acalypha fruticosa* Forssk. | (Bijekar and Gayatri, 2014)* |
|  |  | *Acalypha grandis* Benth. | (Seebaluck et al., 2015)*  (van Valkenburg and Bunyapraphatsara, 2001)*  (World Health Organization (WHO), 2009)* |
|  |  | *Acalypha hispida* Burm. F. | (Bokshi et al., 2012)  (Kirtikar and Basu, 1935)*  (Seebaluck et al., 2015)* |
|  |  | *Acalypha indica* L. | (Das et al., 2005)  (Quattrocchi, 2012)*  (Seebaluck et al., 2015)  (Zahidin et al., 2017)* |
|  |  | *Acalypha manniana* Müll.Arg. | (Hamill et al., 2000)* |
|  |  | *Acalypha polymorpha* Müll.Arg. | (Quattrocchi, 2012)* |
|  |  | *Acalypha psilostachya* Hochst. Ex A.Rich. | (Neuwinger, 2000)  (Quattrocchi, 2012)*  (Schmelzer et al., 2008) |
|  |  | *Acalypha villicaulis* Hochst. Ex A.Rich. | (Quattrocchi, 2012)*  (Schmelzer et al., 2008)* |
|  |  | *Acalypha wilkesiana* Müll.Arg. | (Quattrocchi, 2012)*  (Seebaluck et al., 2015)*  (van Valkenburg and Bunyapraphatsara, 2001)*  (World Health Organization (WHO), 2009)* |
|  | Fever | *Acalypha andringitrensis* Leandri | (Quattrocchi, 2012)*  (Schmelzer et al., 2008)*  (Seebaluck et al., 2015)* |
|  |  | *Acalypha fruticosa* Forssk. | (Hassan-Abdallah et al., 2013)  (Quattrocchi, 2012)*  (Ruffo et al., 2002)*  (Schmelzer et al., 2008)*  (Zahidin et al., 2017)* |
|  |  | *Acalypha indica* L. | (Zahidin et al., 2017)* |
|  |  | *Acalypha radula* Baker | (Quattrocchi, 2012)* |
|  |  | *Acalypha siamensis* Oliv. Ex Gage | (Ng and Songkhla, 2000)  (Quattrocchi, 2012)*  (Seebaluck et al., 2015)*  (van Valkenburg and Bunyapraphatsara, 2001)*  (Wiart et al., 2004) |
|  |  | *Acalypha villicaulis* Hochst. Ex A.Rich. | (Schmelzer et al., 2008)*  (Seebaluck et al., 2015)* |
|  |  | *Acalypha wilkesiana* Müll.Arg. | (Odugbemi, 2008)*  (Mendame et al., 2022)  (van Valkenburg and Bunyapraphatsara, 2001)*  (World Health Organization  (WHO), 2009)* |
|  | Inflammation | *Acalypha allenii* Hutch. | (Quattrocchi, 2012)* |
|  |  | *Acalypha alopecuroidea* Jacq. | (Alonso-Castro et al., 2011)*  (Argueta et al., 1994)*  (Del Rosario Pérez and Weniger, 1988)  (Hernández et al., 2003)  (Madlener et al., 2009)  (Quattrocchi, 2012)*  (Seebaluck et al., 2015)*  (Svačinová, 2011) |
|  |  | *Acalypha arvensis* Poepp. | (Arvigo and Balick, 1993)* |
|  |  | *Acalypha australis* L. | (Dong et al., 1994)  (Quattrocchi, 2012)*  (Seebaluck et al., 2015)*  (van Valkenburg and Bunyapraphatsara, 2001)* |
|  |  | *Acalypha fruticosa* Forssk. | (Bijekar and Gayatri, 2014)*  (Quattrocchi, 2012)*  (Schmelzer et al., 2008)* |
|  |  | *Acalypha grandis* Benth. | (McClatchey, 1996)  (van Valkenburg and Bunyapraphatsara, 2001)* |
|  |  | *Acalypha indica* L. | (Ganesan and Xu, 2017)  (Quattrocchi, 2012)* |
|  |  | *Acalypha lanceolata* Willd. | (Quattrocchi, 2012)* |
|  |  | *Acalypha monostachya* Cav. | (Canales et al., 2005) |
|  |  | *Acalypha multicaulis* Müll.Arg. | (de Albuquerque et al., 2007)* |
|  |  | *Acalypha novoguineensis* Warb. | (van Valkenburg and Bunyapraphatsara, 2001) |
|  |  | *Acalypha phleoides* Cav. | (Alonso-Castro et al., 2011)* |
|  |  | *Acalypha wilkesiana* Müll.Arg. | (Odugbemi, 2008)*  (Quattrocchi, 2012)*  (van Valkenburg and Bunyapraphatsara, 2001)* |
|  | Dizziness | *Acalypha psilostachya* Hochst. Ex A.Rich. | (Neuwinger, 2000)  (Schmelzer et al., 2008)* |
|  | Others | *Acalypha australis* L. | (van Valkenburg and Bunyapraphatsara, 2001)* |
|  |  | *Acalypha indica* L. | (Ganesan and Xu, 2017)  (Quattrocchi, 2012)* |
| **Injuries** | Bites and stings | *Acalypha australis* L. | (Quattrocchi, 2012)*  (van Valkenburg and Bunyapraphatsara, 2001)* |
|  |  | *Acalypha fruticosa* Forssk. | (Bijekar and Gayatri, 2014)*  (Pakia et al., 2003)  (Quattrocchi, 2012)*  (Schmelzer et al., 2008)* |
|  |  | *Acalypha indica* L. | (Cooper, 1842)*  (Dineshkumar et al., 2010)*  (Drury, 1873)*  (Kirtikar and Basu, 1935)*  (Quattrocchi, 2012)*  (Zahidin et al., 2017)* |
|  |  | *Acalypha paniculata* Miq. | (Quattrocchi, 2012)* |
|  |  | *Acalypha villicaulis* Hochs. Ex A.Rich. | (Quattrocchi, 2012)*  (Schmelzer et al., 2008)*  (Seebaluck et al., 2015)* |
|  | Wounds | *Acalypha bipartita* Müll.Arg. | (Tabuti et al., 2003) |
|  |  | *Acalypha fruticosa* Forssk. | (Bijekar and Gayatri, 2014)*  (Hassan-Abdallah et al., 2013)  (Quattrocchi, 2012)*  (Schmelzer et al., 2008)*  (Seebaluck et al., 2015)* |
|  |  | *Acalypha grandis* Benth. | (van Valkenburg and Bunyapraphatsara, 2001)* |
|  |  | *Acalypha hellwigii* Warb. | (Quattrocchi, 2012)* |
|  |  | *Acalypha hispida* Burm. F. | (Bokshi et al., 2012)  (P. Onocha et al., 2011a, 2011b; P. A. Onocha et al., 2011)  (Quattrocchi, 2012)*  (Seebaluck et al., 2015)*  (van Valkenburg and Bunyapraphatsara, 2001)* |
|  |  | *Acalypha indica* L. | (Islam et al., 2019)*  (Kirtikar, 1935)*  (Quattrocchi, 2012)*  (Seebaluck et al., 2015)*  (Selvaraju et al., 2011)  (Dinesh and P, 2010)  (Zahidin et al., 2017) |
|  |  | *Acalypha insulana* Müll.Arg. | (Quattrocchi, 2012)* |
|  |  | *Acalypha langiana* Müll.Arg. | (Quattrocchi, 2012)* |
|  |  | *Acalypha monostachya* Cav. | (Canales et al., 2005) |
|  |  | *Acalypha monoguineensis* Warb. | (Quattrocchi, 2012)*  (Worth and Sakulas, 1987) |
|  |  | *Acalypha siamensis* Oliv. Ex Gage | (Seebaluck et al., 2015)*  (Wiart et al., 2004) |
|  |  | *Acalypha villicaulis* Hochst. Ex A.Rich. | (Quattrocchi, 2012)*  (Schmelzer et al., 2008)*  (Seebaluck et al., 2015)* |
|  | Ulcers, sores | *Acalypha ciliata* Forssk. | (Schmelzer et al., 2008)* |
|  |  | *Acalypha fruticosa* Forssk. | (Schmelzer et al., 2008)* |
|  |  | *Acalypha hellwigii* Warb. | (Quattrocchi, 2012)* |
|  |  | *Acalypha hispida* Burm. F. | (Kirtikar and Basu, 1935)*  (Quattrocchi, 2012)* |
|  |  | *Acalypha indica* L. | (Dineshkumar et al., 2010)*  (Drury, 1873)*  (Kirtikar and Basu, 1935)*  (Todd., 1888) |
|  |  | *Acalypha insulana* Müll.Arg. | (Quattrocchi, 2012)* |
|  |  | *Acalypha lanceolata* Willd. | (Quattrocchi, 2012)* |
|  |  | *Acalypha ornata* Hochst. Ex A.Rich. | (Burkill, 1994)*  (Aboaba et al., 2012) |
|  |  | *Acalypha villicaulis* Hochst. Ex A.Rich. | (Quattrocchi, 2012)*  (Schmelzer et al., 2008)* |
|  |  | *Acalypha volkensii* Pax | (Quattrocchi, 2012)* |
|  | Burns | *Acalypha indica* L. | (Quattrocchi, 2012)* |
|  |  | *Acalypha lanceolata* Willd. | (Quattrocchi, 2012)*  (van Valkenburg and Bunyapraphatsara, 2001)* |
|  | Others | *Acalypha alopecuroidea* Jacq. | (Argueta et al., 1994)*  (Johnson, 2019)*  (Svačinová, 2011) |
|  |  | *Acalypha ciliata* Forssk. | (Quattrocchi, 2012)* |
|  |  | *Acalypha wilkesiana* Müll.Arg. | (Quattrocchi, 2012)* |
| **Poisoning** | - | *Acalypha indica* L. | (Quattrocchi, 2012)*  (Schmelzer et al., 2008)* |
|  |  | *Acalypha psilostachya* Hochst. Ex A.Rich. | (Quattrocchi, 2012)* |
|  |  | *Acalypha wilkesiana* Müll.Arg. | (van Valkenburg and Bunyapraphatsara, 2001)* |
| **Lymphatic system** | - | *Acalypha villicaulis* Hochst. Ex A.Rich. | (Quattrocchi, 2012)*  (Schmelzer et al., 2008)*  (Seebaluck et al., 2015)* |
|  |  | *Acalypha virginica* L. | (Porcher, 1849)*  (Williams, 1849)* |
|  |  | *Acalypha wilkesiana* Müll.Arg. | (Quattrocchi, 2012)*  (van Valkenburg and Bunyapraphatsara, 2001)* |
| **Others** | - | *Acalypha ambigua* Pax | (Quattrocchi, 2012)* |
|  |  | *Acalypha australis* L. | (Quattrocchi, 2012)* |
|  |  | *Acalypha ciliata* Forssk. | (Quattrocchi, 2012)* |
|  |  | *Acalypha fruticosa* Forssk. | (Drury, 1873)*  (Hassan-Abdallah et al., 2013)  (Schmelzer et al., 2008)*  (Seebaluck et al., 2015)* |
|  |  | *Acalypha indica* L. | (Quattrocchi, 2012)*  (Zahidin et al., 2017)* |
|  |  | *Acalypha paniculata* Miq. | (Quattrocchi, 2012)* |
|  |  | *Acalypha platyphylla* Müll.Arg. | (Quattrocchi, 2012)* |
|  |  | *Acalypha siamensis* Oliv. Ex Gage | (Quattrocchi, 2012)* |
|  |  | *Acalypha villicaulis* Hochst. Ex A.Rich. | (Mohagheghzadeh et al., 2006)  (Tabuti et al., 2003) |
|  |  | *Acalypha volkensii* Pax | (Quattrocchi, 2012)* |

1. Veterinary medicine

| **Type of disease** | | **Species** | **References** |
| --- | --- | --- | --- |
| **First classification** | **Second classification** |  |  |
| **Digestive tract** | - | *Acalypha indica* L. | (Eswaran et al., 2013)  (Pragada et al., 2011)  (Seebaluck et al., 2015)* |
| **Infectious and parasitic diseases** | - | *Acalypha indica* L. | (Giday and Teklehaymanot, 2013)  (Seebaluck et al., 2015)* |
| **Respiratory system** | - | *Acalypha indica* L. | (Pandit, 2010)  (Rao et al., 2008)  (Seebaluck et al., 2015)* |
| **Skin** | - | *Acalypha indica* L. | (Kiruba et al., 2006)  (Mallik et al., 2012)  (Seebaluck et al., 2015)* |
| **Injuries** | Wounds | *Acalypha fruticosa* Forssk. | (Schmelzer et al., 2008)* |
|  |  | *Acalypha indica* L. | (Seebaluck et al., 2015)*  (Selvaraju et al., 2011)  (Dinesh and P, 2010) |
| **Others** | - | *Acalypha ambigua* Pax | (Quattrocchi, 2012)* |
|  |  | *Acalypha fruticosa* Forssk. | (Quattrocchi, 2012)* |
|  |  | *Acalypha indica* L. | (Quattrocchi, 2012)*  (van Valkenburg and Bunyapraphatsara, 2001)* |
|  |  | *Acalypha villicaulis* Hochst. Ex A.Rich. | (Quattrocchi, 2012)* |

1. Rituals (ethnobotanical info)

| **Species** | **References** |
| --- | --- |
| *Acalypha ambigua* Pax | (Quattrocchi, 2012)* |
| *Acalypha ciliata* Forssk. | (Quattrocchi, 2012)* |
| *Acalypha echinus* Pax & K.Hoffm. | (Pakia et al., 2003) |
| *Acalypha fruticosa* Forssk. | (Pakia et al., 2003) |
| *Acalypha hellwigii* Warb. | (Quattrocchi, 2012)* |
| *Acalypha insulana* Müll.Arg. | (Quattrocchi, 2012)* |
| *Acalypha neptunica* Müll.Arg. | (Pakia et al., 2003) |
| *Acalypha segetalis* Müll.Arg. | (Quattrocchi, 2012)* |

1. Plaguicides

| **Species** | **References** |
| --- | --- |
| *Acalypha alnifolia* J.G. Klein ex Willd. | (Kamalakannan and Gopinath, 2013)  (Seebaluck et al., 2015)* |
| *Acalypha ciliata* Forssk. | (Quattrocchi, 2012)* |
| *Acalypha fruticosa* Forssk. | (Quattrocchi, 2012)* |
| *Acalypha ornata* Hochst. Ex A.Rich. | (Quattrocchi, 2012)* |
| *Acalypha segetalis* Müll.Arg. | (Quattrocchi, 2012)* |

## *Appendix 2.* Checklist of medicinal uses in experimental studies of *Acalypha* species organised by type of study.

1. *In vitro* studies

| **Type of disease or interest** | **Species** | **References** |
| --- | --- | --- |
| **Antioxidant** | *Acalypha bipartita* Müll.Arg. | (Andabati and Muyonga, 2014) |
|  | *Acalypha diversifolia* Jacq. | (Mosquera et al., 2007) |
|  | *Acalypha fruticosa* Forssk. | (Gopalakrishnan et al., 2010)  (Rajkumar et al., 2010)  (Schmelzer et al., 2008)*  (Seebaluck et al., 2015)* |
|  | *Acalypha guatemalensis* Pax and K.Hoffm. | (Navarro et al., 2003) |
|  | *Acalypha platyphylla* Müll.Arg. | (Mosquera et al., 2007)  (Seebaluck et al., 2015)* |
|  | *Acalypha segetalis* Müll.Arg. | (Seebaluck et al., 2015)*  (Tauseef et al., 2013) |
|  | *Acalypha wilkesiana* Müll.Arg. | (Seebaluck et al., 2015)*  (Tauseef et al., 2013) |
| **Neoplasm** | *Acalypha alopecuroidea* Jacq. | (Madlener et al., 2009)  (Seebaluck et al., 2015)*  (Svačinová, 2011) |
|  | *Acalypha californica* Benth. | (Rascón-Valenzuela et al., 2015) |
|  | *Acalypha fruticosa* Forssk. | (Gopalakrishnan et al., 2010)  (Mothana et al., 2010)  (Rajkumar et al., 2010)  (Seebaluck et al., 2015)* |
|  | *Acalypha grandis* Benth. | (Bradacs et al., 2010) |
|  | *Acalypha hispida* Burm. F. | (Schmelzer et al., 2008)* |
|  | *Acalypha indica* L. | (Sanseera et al., 2012)  (Zahidin et al., 2017)* |
|  | *Acalypha wilkesiana* Müll.Arg. | (Büssing et al., 1999) |
| **Infectious and parasitic diseases** | *Acalypha californica* Benth. | (Robles-Zepeda et al., 2013) |
|  | *Acalypha ciliata* Wall. | (Adesina et al., 2000) |
|  | *Acalypha communis* Pax and K.Hoffm. | (Gutiérrez-Lugo et al., 2002)  (Seebaluck et al., 2015)* |
|  | *Acalypha diversifolia* Jacq. | (Niño et al., 2012) |
|  | *Acalypha fruticosa* Forssk. | (Bama et al., 2013)  (Gopalakrishnan et al., 2010)  (Mothana et al., 2010)  (Thambiraj and Paulsamy, 2011)  (Schmelzer et al., 2008)*  (Seebaluck et al., 2015)* |
|  | *Acalypha grandis* Benth. | (Bradacs et al., 2010) |
|  | *Acalypha guatemalensis* Pax and K.Hoffm. | (Cáceres et al., 1998)  (Navarro et al., 2003) |
|  | *Acalypha hispida* Burm. F. | (Adesina et al., 2000)  (Bokshi et al., 2012)  (P. A. Onocha et al., 2011)  (Schmelzer et al., 2008)*  (Seebaluck et al., 2015)* |
|  | *Acalypha indica* L. | (Chengaiah et al., 2009)  (Govindarajan et al., 2008)  (Krishnaraj et al., 2010)  (Manonmani et al., 2015)  (Mohan et al., 2012)  (Noriko, 2013)  (Perumal et al., 1999)  (Rajaselvam et al., 2012)  (Ranju et al., 2011)  (Selvamani and Balamurugan, 2015)  (Somchit et al., 2010) |
|  | *Acalypha monostachya* Cav. | (Canales et al., 2005) |
|  | *Acalypha ornata* Hochst. Ex A.Rich. | (Emeka et al., 2012)  (Seebaluck et al., 2015)* |
|  | *Acalypha phleoides* Cav. | (Calzada et al., 1998) |
|  | *Acalypha platyphylla* Müll.Arg. | (Niño et al., 2012) |
|  | *Acalypha reflexa* Müll.Arg. | (Bussmann et al., 2010) |
|  | *Acalypha siamensis* Oliv. Ex Gage | (Seebaluck et al., 2015)*  (Wiart et al., 2004) |
|  | *Acalypha wilkesiana* Müll.Arg. | (Adesina et al., 2000)  (Akinyemi et al., 2005)  (Gotep et al., 2010)  (Majekodunmi and Nubani, 2014)  (Oluduro et al., 2011)  (Othman et al., 2011)  (P. A. Onocha et al., 2011, 2011)  (Santiago et al., 2015)  (Seebaluck et al., 2015)* |
| **Locomotor system** | *Acalypha indica* L. | (Jayaprakasam and Ravi, 2013) |
| **Symptoms** | *Acalypha fruticosa* Forssk. | (Gopalakrishnan et al., 2010)  (Seebaluck et al., 2015)* |
| **Injuries** | *Acalypha fruticosa* Forssk. | (Gopalakrishnan et al., 2010)  (Seebaluck et al., 2015)* |
| **Poisoning** | *Acalypha indica* L. | (Rajendran et al., 2010) |

1. *Ex vivo* studies

| **Type of disease** | **Species** | **References** |
| --- | --- | --- |
| **Neoplasm** | *Acalypha fruticosa* Forssk. | (Rajkumar et al., 2010)  (Seebaluck et al., 2015)* |
| **Cardiovascular system** | *Acalypha wilkesiana* Müll.Arg. | (Ezekwesili et al., 2012)  (Seebaluck et al., 2015)* |
| **Digestive tract** | *Acalypha phleoides* Cav. | (Astudillo et al., 2004)  (Seebaluck et al., 2015)* |
|  | *Acalypha wilkesiana* Müll.Arg. | (Ezekwesili et al., 2012)  (Seebaluck et al., 2015)* |
| **Respiratory system** | *Acalypha phleoides* Cav. | (Astudillo et al., 2004) |
| **Others** | *Acalypha wilkesiana* Müll.Arg. | (Makoshi et al., 2016) |

1. *In vivo* studies

| **Type of disease** | **Species** | **References** |
| --- | --- | --- |
| **Antioxidant** | *Acalypha indica* L. | (Priya and Bhaskara Rao, 2016) |
| **Neoplasm** | *Acalypha fruticosa* Forssk. | (Sivakumar et al., 2010) |
|  | *Acalypha hispida* Burm. F. | (P. A. Onocha et al., 2011)  (Seebaluck et al., 2015)* |
| **Cardiovascular system** | *Acalypha wilkesiana* Müll.Arg. | (Ezekwesili et al., 2012)  (C. J. Ikewuchi et al., 2009; Ikewuchi et al., 2011; J. C. Ikewuchi et al., 2009)  (J. C. Ikewuchi et al., 2009)  (Seebaluck et al., 2015)* |
| **Digestive tract** | *Acalypha australis* L. | (Tan et al., 2024) |
|  | *Acalypha communis* Müll.Arg. | (Rajasekaran and Anandan, 2016) |
|  | *Acalypha fimbriata* Schumach. And Thonn. | (Quds et al., 2012)  (Seebaluck et al., 2015)* |
|  | *Acalypha ornata* Hochst. Ex A.Rich. | (Quds et al., 2012)  (Seebaluck et al., 2015)* |
|  | *Acalypha phleoides* Cav. | (Astudillo et al., 2004)  (Seebaluck et al., 2015)* |
|  | *Acalypha wilkesiana* Müll.Arg. | (Quds et al., 2012)  (Seebaluck et al., 2015)* |
| **Urinary system** | *Acalypha indica* L. | (Das et al., 2005)  (Seebaluck et al., 2015)* |
| **Reproductive system** | *Acalypha indica* L. | (Hiremath et al., 1999) |
| **Gynecological problems** | *Acalypha indica* L. | (Hiremath et al., 1999) |
| **Endocrine – metabolic system** | *Acalypha alnifolia* J.G. Klein ex Willd. | (Johnkennedy et al., 2011)  (Seebaluck et al., 2015)* |
|  | *Acalypha indica* L. | (Priya and Bhaskara Rao, 2016) |
| **Locomotor system** | *Acalypha alopecuroidea* Jacq. | (Zavala-Sánchez et al., 2009) |
| **Skin** | *Acalypha wilkesiana* Müll.Arg. | (Oyelami et al., 2003)  (Seebaluck et al., 2015)* |
| **Infectious and parasitic diseases** | *Acalypha fruticosa* Forssk. | (Ireri et al., 2010)  (Mong’are et al., 2013)  (Seebaluck et al., 2015)* |
|  | *Acalypha hispida* Burm. f. | (Bokshi et al., 2012)  (P. Onocha et al., 2011b; P. A. Onocha et al., 2011)  (Seebaluck et al., 2015)* |
|  | *Acalypha wilkesiana* Müll.Arg. | (Udobang et al., 2010) |
| **Symptoms** | *Acalypha alnifolia* J.G. Klein ex Willd. | (Ponnusamy et al., 2013) |
|  | *Acalypha alopecuroidea* Jacq. | (Seebaluck et al., 2015)*  (Zavala-Sánchez et al., 2009) |
|  | *Acalypha fruticosa* Forssk. | (Schmelzer et al., 2008)*  (Seebaluck et al., 2015) |
|  | *Acalypha indica* L. | (Rahman et al., 2010) |
|  | *Acalypha wilkesiana* Müll.Arg. | (Ogbunugafor et al., 2011) |
| **Injuries**  **Poisoning** | *Acalypha indica* L. | (Moorthy et al., 2012)  (Seebaluck et al., 2015)* |
|  | *Acalypha langiana* Müll.Arg. | (Pérez Gutiérrez and Vargas, 2006) |
|  | *Acalypha indica* L. | (Mandal and Khora, 2013)  (Schmelzer et al., 2008)* |
| **Others** | *Acalypha wilkesiana* Müll.Arg. | (Makoshi et al., 2016) |
| **Plaguicides** | *Acalypha ciliata* Forssk. | (Aboaba et al., 2012)  (Seebaluck et al., 2015)* |
|  | *Acalypha gaumeri* Pax and K.Hoffm. | (Aboaba et al., 2012)  (Cruz-Estrada et al., 2013)  (Seebaluck et al., 2015)* |
|  | *Acalypha ornata* Hochst. Ex A.Rich. | (Aboaba et al., 2012)  (Schmelzer et al., 2008)* |

**REFERENCES**

Aboaba, S., Ibrahim, K., Omotoso, O., 2012. Toxicity and mosquito larvicidal activities of the essential oils from the leaves of Acalypha ornata and Acalypha ciliata in southwest Nigeria. J. Vector Borne Dis. 49, 114–6.

Adesina, S.K., Idowu, O., Ogundaini, A.O., Oladimeji, H., Olugbade, T.A., Onawunmi, G.O., Pais, M., 2000. Antimicrobial constituents of the leaves of Acalypha wilkesiana and Acalypha hispida. Phytother. Res. 14, 371–374. https://doi.org/10.1002/1099-1573(200008)14:5<371::AID-PTR625>3.0.CO;2-F

Adsul, Y.D., Mahajan, R.T., Badgujar, S.B., 2013. Ethnobotanical Euphorbian plants of North Maharashtra Region. IOSR J. Pharm. Biol. Sci. 7, 29–35. https://doi.org/10.9790/3008-0712935

Afolayan, A.J., Grierson, D.S., Mbeng, W.O., 2014. Ethnobotanical survey of medicinal plants used in the management of skin disorders among the Xhosa communities of the Amathole District, Eastern Cape, South Africa. J. Ethnopharmacol. 153, 220–232. https://doi.org/10.1016/j.jep.2014.02.023

Aishwarya, S.S., Asmita, A.T., Supriya, D.A., 2024. Acalypha indica: phytochemical constituents, traditional uses and pharmacological properties: a review. Int. J. Pharm. Sci. Res. 15, 1059–1064. https://doi.org/10.13040/IJPSR.0975-8232.15(4).1059-64

Akinyemi, K.O., Oladapo, O., Okwara, C.E., Ibe, C.C., Fasure, K.A., 2005. Screening of crude extracts of six medicinal plants used in South-West Nigerian unorthodox medicine for anti-methicillin resistant Staphylococcus aureus activity. BMC Complement. Altern. Med. 5, 6. https://doi.org/10.1186/1472-6882-5-6

Alonso-Castro, A.J., Villarreal, M.L., Salazar-Olivo, L.A., Gomez-Sanchez, M., Dominguez, F., Garcia-Carranca, A., 2011. Mexican medicinal plants used for cancer treatment: Pharmacological, phytochemical and ethnobotanical studies. J. Ethnopharmacol. 133, 945–972. https://doi.org/10.1016/j.jep.2010.11.055

Andabati, B., Muyonga, J., 2014. Phenolic content and antioxidant activity of selected Ugandan traditional medicinal foods. Afr. J. Food Sci. 8, 427–434. https://doi.org/10.5897/AJFS2014.1136

Argueta, A., Gallardo Vázquez, M.C., Instituto Nacional Indigenista (Mexico) (Eds.), 1994. Atlas de las plantas de la medicina tradicional mexicana, 1. ed. ed, Biblioteca de la medicina tradicional mexicana. Instituto Nacional Indigenista, México, D.F.

Arias, B.T., 2009. Diversidad de usos, prácticas de recolección y diferencias según género y edad en el uso de plantas medicinales en Córdoba, Argentina. Bol. Latinoam. Caribe Plantas Med. Aromáticas 8, 389–401.

Arvigo, R., Balick, M.J., 1993. Rainforest Remedies: One Hundred Healing Herbs of Belize. Lotus Press.

Astudillo, A., Hong, E., Bye, R., Navarrete, A., 2004. Antispasmodic activity of extracts and compounds of Acalypha phleoides Cav. Phytother. Res. 18, 102–106. https://doi.org/10.1002/ptr.1414

Ayele, T.T., 2018. A Review on Traditionally Used Medicinal Plants/Herbs for Cancer Therapy in Ethiopia: Current Status, Challenge and Future Perspectives. Org. Chem. Curr. Res. 07. https://doi.org/10.4172/2161-0401.1000192

Balakrishnan, V., Prema, P., Ravindran, K.C., Robinson, J.P., 2009. Ethnobotanical Studies among Villagers from Dharapuram Taluk, Tamil Nadu, India. Glob. J. Pharmacol. 3, 08–14.

Bama, S., Sankaranarayanan, S., Bama, P., Ramachandran, J., Bhuvaneswari, N., Jayasurya Kingsley, S., 2013. Antibacterial activity of medicinal plants used as ethnomedicine by the traditional healers of Musiri Thaluk, Trichy District, Tamilnadu, India. J. Med. Plants Res. 7, 1452–1460. https://doi.org/10.5897/JMPR12.851

Bandyopadhyay, S., Mukherjee, S.K., 2005. Ethnoveterinary medicine from Koch Bihar district, West Bengal. Indian J. Tradit. Knowl. 4, 456–461.

Beyra, Á., Volpato, G., Godínez, D., Guimarais, M., Álvarez, R., 2004. Estudios etnobotánicos sobre plantas medicinales en la provincia de Camagüey (Cuba). An. Jardín Botánico Madr.

Bhat, R.B., 2013. Plants of Xhosa people in the Transkei region of Eastern Cape (South Africa) with major pharmacological and therapeutic properties. J. Med. Plants Res. 7, 1474–1480. https://doi.org/10.5897/JMPR12. 973

Bijekar, S., Gayatri, M., 2014. Ethanomedicinal properties of euphorbiaceae family-A comprehensive review. Int. J. Phytomedicine 6, 144–156.

Bokshi, B., Siraj, M.A., Ahmed, Md.I., Karmakar, U., Sadhu, S., 2012. Assesnebt if abtunucribuak and cytotoxic activities of ethanolic extract of leaves of Acalypha hispida. Int. J. Pharm. Sci. Res. IJPSR 6, 1705–1708.

Bradacs, G., Maes, L., Heilmann, J., 2010. In vitro cytotoxic, antiprotozoal and antimicrobial activities of medicinal plants from Vanuatu. Phytother. Res. 24, 800–809. https://doi.org/10.1002/ptr.2981

Burkill, H.M., 1994. The Useful Plants of West Tropical Africa: families E-I. Royal Botanic Gardens.

Büssing, A., Stein, G.M., Herterich-Akinpelu, I., Pfüller, U., 1999. Apoptosis-associated generation of reactive oxygen intermediates and release of pro-inflammatory cytokines in human lymphocytes and granulocytes by extracts from the seeds of Acalypha wilkesiana. J. Ethnopharmacol. 66, 301–309. https://doi.org/10.1016/S0378-8741(98)00227-X

Bussmann, R.W., Glenn, A., Sharon, D., 2010. Antibacterial activity of medicinal plants of Northern Peru – can traditional applications provide leads for modern science? IJTK Vol94 Oct. 2010.

Cáceres, A., López, B., González, S., Berger, I., Tada, I., Maki, J., 1998. Plants used in Guatemala for the treatment of protozoal infections. I. Screening of activity to bacteria, fungi and American trypanosomes of 13 native plants1. J. Ethnopharmacol. 62, 195–202. https://doi.org/10.1016/S0378-8741(98)00140-8

Calzada, F., Meckes, M., Cedillo-Rivera, R., Tapia-Contreras, A., Mata, R., 1998. Screening of Mexican Medicinal Plants for Antiprotozoal Activity. Pharm. Biol. 36, 305–309. https://doi.org/10.1076/phbi.36.5.305.4653

Canales, M., Hernández, T., Caballero, J., Vivar, A.R. de, Avila, G., Duran, A., Lira, R., 2005. Informant consensus factor and antibacterial activity of the medicinal plants used by the people of San Rafael Coxcatlán, Puebla, México. J. Ethnopharmacol. 97, 429–439. https://doi.org/10.1016/j.jep.2004.11.013

Carmona, J.A., Gil, R.O., Rodríguez, M.C.A., 2008. Descripción taxonómica, morfológica y etnobotánica de 26 hierbas comunes que crecen en la ciudad de Mérida-Venezuela. Bol. Antropológico 26, 113–129.

Chengaiah, B., KUMAR, K., Muthumanickam, A., SASIKALA, C., MADHUSUDHANA, C., 2009. In-vitro anthelmintic activity of roots of Acalypha indica Linn. Int. J. PharmTech Res. 1.

Cooper, D., 1842. Notes and memoranda on the properties and uses of plants, collected from the Herbarium of the medical officers of the army: kep at the Army Medical Museum, Fort Pitt, Chatham. The Lancet 39, 164–167. https://doi.org/10.1016/S0140-6736(02)76517-3

Cruz-Estrada, A., Gamboa-Angulo, M., Borges-Argáez, R., Ruiz-Sánchez, E., 2013. Insecticidal effects of plant extracts on immature whitefly Bemisia tabaci Genn. (Hemiptera: Aleyroideae). Electron. J. Biotechnol. 16, 6–6. https://doi.org/10.2225/vol16-issue1-fulltext-6

Das, A., Ahmed, F., Biswas, N., Dev, S., Masud, M.M., 2005. Diuretic Activity of Acalypha indica. Dhaka Univ. J. Pharm. Sci. 4, 77. https://doi.org/10.3329/dujps.v4i1.204

de Albuquerque, U.P., de Medeiros, P.M., de Almeida, A.L.S., Monteiro, J.M., de Freitas Lins Neto, E.M., de Melo, J.G., dos Santos, J.P., 2007. Medicinal plants of the *caatinga* (semi-arid) vegetation of NE Brazil: A quantitative approach. J. Ethnopharmacol. 114, 325–354. https://doi.org/10.1016/j.jep.2007.08.017

Del Rosario Pérez, R., Weniger, B., 1988. Seminario Tramil 3, in: Seminario Tramil 3. Presented at the Seminario Tramil, La Habana, Cuba, p. 16.

Dinesh, V., P, S.P., 2010. Traditional Uses of Plants in Indigenous Folklore of Nizamabad District, Andhra Pradesh, India. Ethnobot. Leafl. 2010.

Dineshkumar, B., Vigneshkumar, P., Bhuvaneshwaran, S.P., Mitra, A., 2010. Phyto-pharmacology of Acalypha indica: A Review. Int. J. Biosci. Altern. Holist. Med. 1, 27–32.

Dlisani, P.B., Bhat, R.B., 1999. Traditional Health Practices in Transkei with Special Emphasis on Maternal and Child Health. Pharm. Biol. 37, 32–36. https://doi.org/10.1076/phbi.37.1.32.6316

Dong, W., Lin, Z., Sun, H., 1994. A new compound from Acalypha australis. Acta Bot. Yunanica 16, 413–416.

Drury, H., 1873. The Useful Plants of India: With Notices of Their Chief Value in Commerce, Medicine, and the Arts. Higginbotham.

Dymock, W., 1884. The Vegetable Materia Medica of Western India. Education Society’s Press.

Emeka, P.M., Badger-Emeka, L.I., Fateru, F., 2012. In vitro antimicrobial activities of Acalypha ornate leaf extracts on bacterial and fungal clinical isolates. J. Herb. Med. 2, 136–142. https://doi.org/10.1016/j.hermed.2012.09.001

Eswaran, S., Boomibalagan, P., Rathinavel, S., 2013. Ethnoveterinary Medicinal Practices of the Villagers of Usilampatti Taluk of Madurai District, India. Int. J. Bot. 9, 37–43. https://doi.org/10.3923/ijb.2013.37.43

Ezekwesili, C., Ghasi, S., Nwodo, O., 2012. Evaluation of the blood pressure lowering activity of leaf extract of acalypha torta Muell. Afr. J. Pharm. Pharmacol. 6, 3235–3241. https://doi.org/10.589/AJPP12112

Ganesan, K., Xu, B., 2017. Ethnobotanical studies on folkloric medicinal plants in Nainamalai, Namakkal District, Tamil Nadu, India. Trends Phytochem. Res. 1, 153–168.

Gbolade, A., 2012. Ethnobotanical study of plants used in treating hypertension in Edo State of Nigeria. J. Ethnopharmacol. 144, 1–10. https://doi.org/10.1016/j.jep.2012.07.018

Giday, M., Teklehaymanot, T., 2013. Ethnobotanical study of plants used in management of livestock health problems by Afar people of Ada’ar District, Afar Regional State, Ethiopia. J. Ethnobiol. Ethnomedicine 9, 8. https://doi.org/10.1186/1746-4269-9-8

Gopalakrishnan, S., Saroja, K., Jeyaseelan, D.E., 2010. Chemical investigation of aerial parts of Acalypha fruticosa forssk. Sch. Res. Libr. 2, 383–389.

Gordon, C.N., 2017. People and protected areas : natural resource harvesting as an approach to support rural communities surrounding Majete Wildlife Reserve, Southern Malawi : a case study. Stellenbosch : Stellenbosch University.

Gotep, J., Agada, Gbise, E., Chollom, S., 2010. Antibacterial Activity of Ethanolic Extract of Acalypha wilkesiana Leaves Growing in Jos, Plateau State, Nigeria. Malays. J. Microbiol. 6, 69–74. https://doi.org/10.21161/mjm.21309

Govindarajan, M., Jebanesan, A., Reetha, D., Amsath, R., Pushpanathan, T., Samidurai, K., 2008. Antibacterial activity of Acalypha indica L. Eur. Rev. Med. Pharmacol. Sci. 12, 299–302.

Gutiérrez-Lugo, M.-T., Singh, M.P., Maiese, W.M., Timmermann, B.N., 2002. New Antimicrobial Cycloartane Triterpenes from Acalypha communis. J. Nat. Prod. 65, 872–875. https://doi.org/10.1021/np020044g

Hamill, F.A., Apio, S., Mubiru, N.K., Mosango, M., Bukenya-Ziraba, R., Maganyi, O.W., Soejarto, D.D., 2000. Traditional herbal drugs of southern Uganda, I. J. Ethnopharmacol. 70, 281–300. https://doi.org/10.1016/S0378-8741(00)00180-X

Hassan-Abdallah, A., Merito, A., Hassan, S., Aboubaker, D., Djama, M., Asfaw, Z., Kelbessa, E., 2013. Medicinal plants and their uses by the people in the Region of Randa, Djibouti. J. Ethnopharmacol. 148, 701–713. https://doi.org/10.1016/j.jep.2013.05.033

Hedberg, I., Hedberg, O., Madati, P.J., Mshigeni, K.E., Mshiu, E.N., Samuelsson, G., 1982. Inventory of plants used in traditional medicine in Tanzania. I. Plants of the families acanthaceae-cucurbitaceae. J. Ethnopharmacol. 6, 29–60. https://doi.org/10.1016/0378-8741(82)90070-8

Hernández, T., Canales, M., Avila, J.G., Duran, A., Caballero, J., Vivar, A.R. de, Lira, R., 2003. Ethnobotany and antibacterial activity of some plants used in traditional medicine of Zapotitlán de las Salinas, Puebla (México). J. Ethnopharmacol. 88, 181–188. https://doi.org/10.1016/S0378-8741(03)00213-7

Hiremath, S.P., Rudresh, K., Badami, S., Patil, S.B., Patil, S.R., 1999. Post-coital antifertility activity of Acalypha indica L. J. Ethnopharmacol. 67, 253–258. https://doi.org/10.1016/S0378-8741(98)00213-X

House, P., Lagos-Witte, S., Torres, C., 1989. Manual popular de 50 plantas medicinales de Honduras. Editorial Guaymuras.

Hurtado Rico, N.E., Rodríguez Jiménez, C., Aguilar Contreras, A., Hurtado Rico, N.E., Rodríguez Jiménez, C., Aguilar Contreras, A., 2006. Estudio cualitativo y flora medicinal del municipio de Copándaro de Galeana, Michoacán, México. Polibotánica 21–50.

Hyams, C.W., 1898. Medicinal Plants which Have Been Collected and Used in North Carolina. North Carolina Agricultural Experiment Station.

Ichikawa, M., 1987. A Preliminary Report on the Ethnobotany of the Suiei Dorobo in Northern Kenya. Afr. Study Monogr. 7, 1–52.

Ignacimuthu, S., Ayyanar, M., Sivaraman K, S., 2006. Ethnobotanical investigations among tribes in Madurai District of Tamil Nadu (India). J. Ethnobiol. Ethnomedicine 2, 25. https://doi.org/10.1186/1746-4269-2-25

Ikewuchi, C.J., Ikewuchi, C.C., Onwuka, C.F., 2009. Acalypha wilkesiana Muell Arg Induced Diuresis in Salt-Loaded Rats: Implications for the Management of Edema, Obesity and Hypertension. J. Appl. Sci. Environ. Manag. 13. https://doi.org/10.4314/jasem.v13i4.55409

Ikewuchi, J., Ikewuchi, C., Onyeike, E.N., Uwakwe, A., 2010. Nutritional Potential of the Leaves of Acalypha wilkesiana ‘Godseffiana’ Muell Arg. J. Appl. Sci. Environ. Manag. 14. https://doi.org/10.4314/jasem.v14i3.61454

Ikewuchi, J.C., Ikewuchi, C.C., Eriyamremu, G.E., 2009. Effect of Acalypha wilkesiana Muell Arg on the Blood Pressure and Aorta Contractility of Salt-Loaded Rats. . Number 10.

Ikewuchi, J.C., Onyeike, E.N., Uwakwe, A.A., Ikewuchi, C.C., 2011. Effect of aqueous extract of the leaves of Acalypha wilkesiana ‘Godseffiana’ Muell Arg (Euphorbiaceae) on the hematology, plasma biochemistry and ocular indices of oxidative stress in alloxan induced diabetic rats. J. Ethnopharmacol. 137, 1415–1424. https://doi.org/10.1016/j.jep.2011.08.015

Ireri, L.N., Kongoro, J., Ngure, P.C., Mutai, C., Lang’at, B., Tonui, W., Kimutai, A., Mucheru, O., 2010. The potential of the extracts of Tagetes minuta Linnaeus (Asteraceae), Acalypha fruticosa Forssk (Euphorbiaceae) and Tarchonanthus camphoratus L. (Compositae) against Phlebotomus duboscqi Neveu Lemaire (Diptera: Psychodidae), the vector for Leishmania major Yakimoff and Schokhor.

Islam, M.S., Ara, H., Ahmad, K.I., Uddin, M.M., 2019. A review on medicinal uses of different plants of Euphorbiaceae family. Univers. J. Pharm. Res. https://doi.org/10.22270/ujpr.v4i1.236

Jayaprakasam, R., Ravi, T.K., 2013. Evaluation of anti arthitic activity of the root extract of Acalypha indica Linn. Using in vitro techniques. Int. J. Phytopharm. 2. https://doi.org/10.7439/ijpp.v2i6.36

Johnkennedy, N., Adamma, E., Nnedimma, N.C., 2011. Hypolipidemic effects of aqueous extract ofAcalypha capitata leaves in rats fed on high cholesterol diet. Asian Pac. J. Trop. Biomed. 1, S183–S185. https://doi.org/10.1016/S2221-1691(11)60152-4

Johnson, T., 2019. CRC Ethnobotany Desk Reference. CRC Press, Boca Raton. https://doi.org/10.1201/9781351070942

Kamalakannan, S., Gopinath, C., 2013. Interaction of Metathizium anisopliae and Acalypha alnifolia on the mosquitocidal and IGR activity of Dengue vector, Aedes Aegypti (L.) (Culicidae: Diptera: Insecta). Int. J. Adv. Biol. Res. 3, 24–30.

Khan, M.R., 2001. Antibacterial Activity of Some Tanzanian Medicinal Plants. Pharm. Biol. 39, 206–212. https://doi.org/10.1076/phbi.39.3.206.5927

Kirtikar, K.R., 1935. Indian Medicinal Plants: By K.R. Kirtikar, B.D. Basu, and An I.C.S. In 4 volumes, 2nd ed. Edited, revised, enlarged, and mostly rewritten by E. Blatter, J.F. Caius, and K.S. Mhaskar. ed. Lalit Mohan Basu, Allahabad.

Kirtikar, K.R., Basu, B.D., 1935. Indian medicinal plants Vol_3.

Kiruba, S., Jeeva, S., Dhas, S.S.M., 2006. Enumeration of ethnoveterinary plants of Cape Comorin, Tamil Nadu. Indian J. Tradit. Knowl. 5, 576–578.

Kovendan, K., Murugan, K., Mahesh Kumar, P., Thiyagarajan, P., John William, S., 2013. Ovicidal, repellent, adulticidal and field evaluations of plant extract against dengue, malaria and filarial vectors. Parasitol. Res. 112, 1205–1219. https://doi.org/10.1007/s00436-012-3252-8

Krishnaraj, C., Jagan, E.G., Rajasekar, S., Selvakumar, P., Kalaichelvan, P.T., Mohan, N., 2010. Synthesis of silver nanoparticles using Acalypha indica leaf extracts and its antibacterial activity against water borne pathogens. Colloids Surf. B Biointerfaces 76, 50–56. https://doi.org/10.1016/j.colsurfb.2009.10.008

Kumar, D., Kumar, A., Prakash, O., 2012. Potential antifertility agents from plants: A comprehensive review. J. Ethnopharmacol. 140, 1–32. https://doi.org/10.1016/j.jep.2011.12.039

Lakshminarayana, V.L., Narasimharao, G.M., 2013. Folk medicines for treating Livestock in Vizianagaram and Srikakulam Districts, Andhra Pradesh, India. Int. J. Adv. Res. Sci. Teechnology 2, 142–146.

Madlener, S., Svacinová, J., Kitner, M., Kopecky, J., Eytner, R., Lackner, A., Vo, T.P.N., Frisch, R., Grusch, M., De Martin, R., Dolezal, K., Strnad, M., Krupitza, G., 2009. In vitro anti-inflammatory and anticancer activities of extracts of Acalypha alopecuroidea (Euphorbiaceae). Int. J. Oncol. 35, 881–891. https://doi.org/10.3892/ijo_00000403

Majekodunmi, S.O., Nubani, S.E., 2014. Formulation of Acalypha wilkesiana Muell. Arg. ethanol leaf extract into creams for the treatment of microbial skin infections. Int. J. Pharm. Sci. Invent. 3, 45–53.

Makoshi, M.S., Oladipo, O.O., Gotep, J.G., Forcados, G.E., Shu, M.L., Chinyere, C.N., Yusuf, H.B., Akanbi, B.O., Samuel, A.L., Ozele, N., Dogonyaro, B.B., Atiku, A.A., Ahmed, M.S., Nduaka, C., 2016. Safety evaluation of Acalypha wilkesiana in albino rats and BHK-21 cell line. Comp. Clin. Pathol. 25, 543–548. https://doi.org/10.1007/s00580-016-2224-2

Mallik, B.K., Panda, T., Padhy, R.N., 2012. Ethnoveterinary practices of aborigine tribes in Odisha, India. Asian Pac. J. Trop. Biomed. 2, S1520–S1525. https://doi.org/10.1016/S2221-1691(12)60447-X

Mandal, N., Khora, S.S., 2013. Ameliorative action of aqueous extract of Acalypha indica against puffer fish Lagocephalus lunaris induced toxicity. Int. J. Drug Dev. Res. 5, 0–0.

Manonmani, P., Ramar, M., Geetha, N., Valan Arasu, M., Erusan, R., Ramaiah, Dr.M., Sowmiya, J., 2015. Synthesis of silver nanoparticles using natural products from Acalypha indica (kuppaimeni) and Curcuma longa (tumeric) on antimicrobial activities. Int. J. Pharm. Res. Bio Sci. 4, 2277–8713.

McClatchey, W., 1996. The ethnopharmacopoeia of Rotuma. J. Ethnopharmacol. 50, 147–156. https://doi.org/10.1016/0378-8741(95)01343-1

McLaughlin, S.P., 1994. Floristic plant geography: the classification of floristic areas and floristic elements. Prog. Phys. Geogr. Earth Environ. 18, 185–208. https://doi.org/10.1177/030913339401800202

Mendame, W.L.M., Mintsa, B.A.E., Nguema, A.-M.N., Pambo, A.B.P., Ibrahim, 2022. Ethnobotanical study of Acalypha wilkesiana (Euphorbiaceae), a plant used in the treatment of arterial hypertension in Oyem in northern Gabon. J. Pharmacogn. Phytochem. 11, 141–145.

Mohagheghzadeh, A., Faridi, P., Shams-Ardakani, M., Ghasemi, Y., 2006. Medicinal smokes. J. Ethnopharmacol. 108, 161–184. https://doi.org/10.1016/j.jep.2006.09.005

Mohan, C., Dinakar, S., Thirupathi, A., Elayaraja, R., Sathiyapriya, B., 2012. Phytochemical, GC-MS analysis and Antibacterial activity of a Medicinal Plant Acalypha indica. Int. J. Pharm Tech Res. 4, 974–4304.

Mong’are, S., Ng’ang’a, Z., Maranga, R., Osiemo, Z., Ngure, P., Ngumbi, P., Tonui, W., 2013. Effect of leaf crude extracts of Tarchonanthus camphoratus (Asteraceae), Acalypha fruticosa (Fabaceae) and Tagetes minuta (Asteraceae) on fecundity of Phlebotomus duboscqi. Sci. Conf. Proc. 0.

Moorthy, G., Ponrasu, T., Krithika, R., Iyappan, K., Gayathri, V.S., Suguna, L., 2012. Topical application of Acalypha indica accelerates rat cutaneous wound healing by up-regulating the expression of Type I and III collagen. J. Ethnopharmacol. 142, 14–22. https://doi.org/10.1016/j.jep.2012.04.005

Moreno-Salazar, S.F., Robles-Zepeda, R.E., Johnson, D.E., 2008. Plant folk medicines for gastrointestinal disorders among the main tribes of Sonora, Mexico. Fitoterapia 79, 132–141. https://doi.org/10.1016/j.fitote.2007.07.009

Moshi, M.J., Kagashe, G.A.B., Mbwambo, Z.H., 2005. Plants used to treat epilepsy by Tanzanian traditional healers. J. Ethnopharmacol. 97, 327–336. https://doi.org/10.1016/j.jep.2004.11.015

Mosquera, O.M., Correa, Y.M., Buitrago, D.C., Niño, J., 2007. Antioxidant activity of twenty five plants from Colombian biodiversity. Mem. Inst. Oswaldo Cruz 102, 631–634. https://doi.org/10.1590/S0074-02762007005000066

Mothana, R.A.A., Abdo, S.A.A., Hasson, S., Althawab, F.M.N., Alaghbari, S.A.Z., Lindequist, U., 2010. Antimicrobial, Antioxidant and Cytotoxic Activities and Phytochemical Screening of Some Yemeni Medicinal Plants. Evid. Based Complement. Alternat. Med. 7, 323–330. https://doi.org/10.1093/ecam/nen004

Navarro, M.C., Montilla, M.P., Cabo, M.M., Galisteo, M., Cáceres, A., Morales, C., Berger, I., 2003. Antibacterial, antiprotozoal and antioxidant activity of five plants used in Izabal for infectious diseases. Phytother. Res. 17, 325–329. https://doi.org/10.1002/ptr.1134

Ndhlovu, P.T., Omotayo, A.O., Otang-Mbeng, W., Aremu, A.O., 2021. Ethnobotanical review of plants used for the management and treatment of childhood diseases and well-being in South Africa. South Afr. J. Bot. 137, 197–215. https://doi.org/10.1016/j.sajb.2020.10.012

Neuwinger, D., 2000. African traditional medicine: a dictionary of plant use and applications with supplement : search system for diseases. Medpharm Scientific Publishers, Stuttgart.

Ng, L.T., Songkhla, B.N., 2000. Acalypha siamensis Oliv. ex Gage [Internet] Record from Proseabase, in: Van der Vossen, H.A.M., Wessel, M. (Eds.), PROSEA (Plant Resources of South-East Asia). PROSEA Foundation, Bogor, Indonesia.

Niño, J., Mosquera, O.M., Correa, Y.M., 2012. Antibacterial and antifungal activities of crude plant extracts from Colombian biodiversity. Rev. Biol. Trop. 60, 1535–1542.

Noriko, N., 2013. Potensi Daun Teh (Camellia sinensis) dan Daun Anting-anting Acalypha indica L. dalam Menghambat Pertumbuhan Salmonella typhi. J. Al-AZHAR Indones. SERI SAINS DAN Teknol. 2, 104–110. https://doi.org/10.36722/sst.v2i2.131

Odugbemi, T., 2008. A Textbook of Medicinal Plants from Nigeria. Tolu Odugbemi.

Ogbunugafor, H.A., Okpuzor, J., Igwo-Ezikpe, M.N., Ezekwesili, C.N., Nebedum, J.O., Ekechi, A.C., A. O, O., 2011. Anti-inflammatory medicinal plants: The effect of three species on serum antioxidant system and lipids levels in rats. NISEB J. 11.

Oliver-Bever, B., 1986. Medicinal Plants in Tropical West Africa. Cambridge University Press.

Oluduro, A.O., Bakare, M.K., Omoboye, O.O., Dada, C.A., Olatunji, C.I., 2011. Antibacterial Effect of Extracts of Acalypha Wilkesiana on Gastrointestinal Tract Pathogens and Bacteria Causing Skin Infection in Neonates. Ife J. Sci. 13, 371–380.

Onocha, P., Oloyede, G., Afolabi, O.O., 2011a. Chemical Composition, Cytotoxicity and Antioxidant Activity of Essential Oils of Acalypha hispida Flowers. Int. J. Pharmacol. 7. https://doi.org/10.3923/ijp.2011.144.148

Onocha, P., Oloyede, G., Dosumu, O., Ali, M., 2011b. Antileishmaniasis and Phytotoxicity of three Nigerian Acalypha species. Adv. Appl. Sci. Res. 3, 1–5.

Onocha, P.A., Oloyede, G.K., Afolabi, Q.O., 2011. Phytochemical investigation, cytotoxicity and free radical scavenging activities of non-polar fractions of Acalypha hispida (leaves and twigs). EXCLI J. 10, 1–8.

Othman, M., Loh, H.S., Wiart, C., Khoo, T.J., Lim, K.H., Ting, K.N., 2011. Optimal methods for evaluating antimicrobial activities from plant extracts. J. Microbiol. Methods 84, 161–166. https://doi.org/10.1016/j.mimet.2010.11.008

Oyelami, O.A., Onayemi, O., Oladimeji, F.A., Ogundaini, A.O., Olugbade, T.A., Onawunmi, G.O., 2003. Clinical evaluation of Acalypha ointment in the treatment of superﬁcial fungal skin diseases. Phytother. Res. 17, 555–557. https://doi.org/10.1002/ptr.1161

Pakia, M., Cooke, J.A., van Staden, J., 2003. The ethnobotany of the Midzichenda tribes of the coastal forest areas in Kenya: 2. Medicinal plant uses. South Afr. J. Bot. 69, 382–395. https://doi.org/10.1016/S0254-6299(15)30321-5

Pandit, P.K., 2010. Inventory of ethhno veterinary medicinal plants of Jhargram division, West Bengal, India. Indian For. 136, 1183–1194.

Pérez Gutiérrez, R.M., Vargas, R.S., 2006. Evaluation of the wound healing properties of Acalypha langiana in diabetic rats. Fitoterapia 77, 286–289. https://doi.org/10.1016/j.fitote.2006.03.011

Perumal, S.R., Ignacimuthu, S., Raja, D.P., 1999. Preliminary screening of ethnomedicinal plants from India. J. Ethnopharmacol. 66, 235–240. https://doi.org/10.1016/S0378-8741(99)00038-0

Ponnusamy, R., Thangaraj, P., Krishnan, S., 2013. Quantification of phenolic compounds, in vitro antioxidant analysis and screening of chemical compounds using GC-MS in Acalypha alnifolia klein ex willd. - A leafy vegetable. Int. J. Pharma Bio Sci. 4, B973–B986.

Porcher, F.P., 1849. Report on the indigenous medicinal plants of South Carolina. American Medical Association, Philadelphia.

Pragada, R., Vangepurapu, V., Ethadi, S., Dasari, P., 2011. Phytochemical investigation and in vitro Anti Oxidant, Anti Microbial activity of different fractions of Acalypha indica linn. Int. J. Pharm. Pharm. Sci. 3, 314–317.

Priya, C.L., Bhaskara Rao, K.V., 2016. Postprandial Antihyperglycemic And Antioxidant Activities of Acalypha indica Linn Stem Extract: An In-vivo Study. Pharmacogn. Mag. 12, S475–S481. https://doi.org/10.4103/0973-1296.191461

Quattrocchi, U., 2012. CRC World Dictionary of Medicinal and Poisonous Plants: Common Names, Scientific Names, Eponyms, Synonyms, and Etymology (5 Volume Set). CRC Press.

Quds, T., Ahmed, S., Ali, M.S., Onocha, P.A., Azhar, I., 2012. Antiemetic activity of Acalypha fimbriata Schumach. & Thonn., Acalypha ornata Hochst., and Acalypha wilkesiana cv. godseffiana Muell Arg. Phytopharmacology 3, 335–340.

Ragunathan, M., Solomon, M., 2012. The study of spiritual remedies in orthodox rural churches and traditional medicinal practice in Gondar Zuria district, Northwestern Ethiopia. Pharmacogn. J. 1, 178–183.

Rahman, M.A., Bachar, S.C., Rahmatullah, M., 2010. Analgesic and antiinflammatory activity of methanolic extract of Acalypha indica Linn. Pak. J. Pharm. Sci. 23, 256–258.

Rajasekaran, S., Anandan, R., 2016. Phytochemical and pharmacological evaluation of Acalypha communis Müll. Arg. for their hepatoprotective activity. Asian J. Pharm. Clin. Res. 94–97.

Rajaselvam, J., Benila smily, Meena, R., 2012. In Vitro Study of Antimicrobial Activity of Acalypha Indica Linn. Extract. Int. J. Pharma Sci. Res. 3. https://doi.org/10.2174/2210289201304020057

Rajendran, K., Shirwaikar, A., Mehta, M., Bharathi, R., 2010. In vitro and in vivo anti-snake venom (Daboia russelli) studies on various leaf extracts of Acalypha indica Linn. Int. J. Phytomedicine 2, 217–220.

Rajkumar, V., Guha, G., Ashok Kumar, R., 2010. Therapeutic potential of Acalypha fruticosa. Food Chem. Toxicol. 48, 1709–1713. https://doi.org/10.1016/j.fct.2010.03.050

Raleigh, N.O., 1898. Medicinal plants which have been collected and used in North Carolina.

Ramathal, D.C., Ngassapa, O.D., 2001. Medicinal Plants Used by Rwandese Traditional Healers in Refugee Camps in Tanzania. Pharm. Biol. 39, 132–137. https://doi.org/10.1076/phbi.39.2.132.6251

Ramya, S., 2008. Ethnomedicinal Perspectives of Botanicals used by Malayali Tribes in Vattal Hills of Dharmapuri (TN), India. Ethnobot. Leafl. 2008.

Ranju, G., Sutar, N., Saroj, K.P., Vishesh, K.P., Shailendr, P., 2011. In vitro anthelmintic activity of Acalypha indica leaves extracts. Int. J. Res. Ayurveda Pharm. 2, 247–249.

Rao, M.L.S., Varma, Y.N.R., Vijaykumar, 2008. Ethno-veterinary Medicinal Plants of the Catchments Area of the River Papagni in the Chittor and Ananthapur Districts of Andhra Pradesh, India. Ethnobot. Leafl. 2008, 26.

Rascón-Valenzuela, L., Jiménez-Estrada, M., Velázquez-Contreras, C., Garibay-Escobar, A., Vilegas, W., Campaner, L., Coqueiro, A., Robles-Zepeda, R.E., 2015. Chemical composition and antiproliferative activity of Acalypha californica. Ind. Crops Prod. 69, 48–54. https://doi.org/10.1016/j.indcrop.2015.02.004

Revathi, P., Parimelazhagan, T., Manian, S., 2013. Quantification of phenolic compounds, in vitro antioxidant analysis and screening of chemical compounds using GC-MS in Acalypha alnifolia klein ex willd. - A leafy vegetable. Int. J. Pharma Bio Sci. 4, 973–986.

Robles-Zepeda, R.E., Coronado-Aceves, E.W., Velázquez-Contreras, C.A., Ruiz-Bustos, E., Navarro-Navarro, M., Garibay-Escobar, A., 2013. In vitro anti-mycobacterial activity of nine medicinal plants used by ethnic groups in Sonora, Mexico. BMC Complement. Altern. Med. 13, 329. https://doi.org/10.1186/1472-6882-13-329

Ruffo, C., Birnie, A., Tengnäs, B., 2002. Edible wild plants of Tanzania, Regional Land Management Unit (RELMA). Tech. Handb. Ser. 27, 766–767.

Sabrina, K., Hladik, C.M., Haxaire, C., 2005. Ethnomedicinal and bioactive properties of plants ingested by wild chimpanzees in Uganda. J. Ethnopharmacol. 101, 1–15. https://doi.org/10.1016/j.jep.2005.03.024

Sanseera, D., Niwatananun, W., Liawruangrath, B., Liawruangrath, S., Baramee, A., Trisuwan, K., Pyne, S., 2012. Antioxidant and anticancer activities from aerial parts of Acalypha indica Linn. Fac. Sci. Med. Health - Pap. Part A 11, 157–168.

Santiago, C., Lim, K.-H., Loh, H.-S., Ting, K.N., 2015. Prevention of cell-surface attachment and reduction of penicillin-binding protein 2a (PBP2a) level in methicillin-resistant Staphylococcus aureus biofilms by Acalypha wilkesiana. BMC Complement. Altern. Med. 15, 79. https://doi.org/10.1186/s12906-015-0615-6

Schmelzer, Gaby H., Gurib-Fakim, A., Schmelzer, Gabriella Harriet, Foundation, P., 2008. Medicinal Plants. PROTA.

Seebaluck, R., Gurib-Fakim, A., Mahomoodally, F., 2015. Medicinal plants from the genus Acalypha (Euphorbiaceae)–A review of their ethnopharmacology and phytochemistry. J. Ethnopharmacol. 159, 137–157. https://doi.org/10.1016/j.jep.2014.10.040

Selvamani, S., Balamurugan, S., 2015. Antibacterial and antifungal activities of different organic solvent extracts of Acalypha indica (Linn.). Asian J. Plant Sci. Res. 5, 52–55.

Selvaraju, A., Ayyanar, M., Rathinakumar, S.S., Sekar, T., 2011. Plants used in ethno-veterinary medicine by malayali tribals in Salem district of Tamil Nadu, India. Med. Plants - Int. J. Phytomedicines Relat. Ind. 3, 209–215.

Senthilkumar, M., Gurumoorthi, P., Janardhanan, K., 2006. Some medicinal plants used by Irular, the tribal people of Marudhamalai hills, Coimbatore, Tamil Nadu. Nat. Prod. Radiance 5, 382–388.

Sivakumar, T., Murthi, M.N.V., Kumutha, P., 2010. Evaluation of anti-tumor and anti-oxidant activity of Acalypha fruticosa in Ehrlich’s Ascites Carcinoma bearing Swiss albino mice. Res. J. Pharm. Biol. Chem. Sci. 1, 191–199.

Somchit, M.N., Rashid, R., Abdullah, A.S., Zuraini, A., Zakaria, Z.A., Sulaiman, M., Arifah, A.K., Mutalib, A., 2010. In vitro antimicrobial activity of leaves of Acalypha indica Linn (Euphorbiaceae). Afr. J. Microbiol. Res.

Sudheer, M., Narayanasamy, A., Anitha, M., 2019. Ethnobotanical study of medicinal plants used by the Irula tribes of Sodungananjanallur village, Karamadai forest beat, Coimbatore district, western ghats range of India. JETIR 6.

Svačinová, J., 2011. PALACKÝ UNIVERSITY IN OLOMOUC FACULTY OF SCIENCE.

Tabuti, J.R.S., Lye, K.A., Dhillion, S.S., 2003. Traditional herbal drugs of Bulamogi, Uganda: plants, use and administration. J. Ethnopharmacol. 88, 19–44. https://doi.org/10.1016/S0378-8741(03)00161-2

Tan, T., Chen, Q., Chen, P., Li, S., Hu, W., Yang, T., Jia, Y., 2024. Zhili decoction ameliorates ulcerative colitis by modulating gut microbiota and related metabolite, and inhibiting the TLR4/NF-κB/NLRP3 pathway. Front. Pharmacol. 15. https://doi.org/10.3389/fphar.2024.1481273

Tauseef, S., Ali, M.S., Ahmed, A., Ali, M.I., Ahmed, Z., Sherwani, S.K., Ahmed, G., Onocha, P.A., Joseph, N., Waffo, A.F.K., Tauseef, F., 2013. In vitro Antioxidant activity analysis of five medicinally important plants. J. Pharmacogn. Phytochem. 2, 183–188.

Thambiraj, J., Paulsamy, S., 2011. Antimicrobial screening of stem extract of the folklore medicinal plant, Acalypha fruticosa forssk. Int. J. Pharm. Pharm. Sci. 3, 285–287.

Todd., A.M., 1888. The treatment and sitillation of peppermint plants. Am. J. Pharm. 60.

Udobang, J.A., Nwafor, P.A., Okokon, J.E., 2010. Analgesic and antimalarial activities of crude leaf extract and fractions of Acalypha wilkensiana. J. Ethnopharmacol. 127, 373–378. https://doi.org/10.1016/j.jep.2009.10.028

van Valkenburg, J., Bunyapraphatsara, N., 2001. Medicinal and poisonous plants 2. Leiden Backhuys Publ. 2001 - Plant Resour. South-East Asia 12 2 - ISBN 90-5782-099-4 12, 784.

Venkateshwarlu, G., Sridhar, C.H., 2017. Folklore uses of Acalypha indica in Ayurveda system. World J. Pharm. Res. 6, 248–250.

Wiart, C., Hannah, A., Yassim, M., Hamimah, H., Sulaiman, M., 2004. Antimicrobial activity of Acalypha siamensis Oliv. ex Gage. J. Ethnopharmacol. 95, 285–286. https://doi.org/10.1016/j.jep.2004.07.014

Williams, S.W., 1849. Report on the indigenous medical botany of Massachusetts. Philadelphia.

World Health Organization (WHO), 2009. Medicinal Plants in Papua New Guinea. World Health Organization, Geneva, Switzerland.

Worth, D.H., Sakulas, H., 1987. Medicinal Plants of the Morobe Province, Papua New Guinea Part IV The Snake River Valley. Int. J. Crude Drug Res. 25, 204–208. https://doi.org/10.3109/13880208709055193

Zahidin, N.S., Saidin, S., Zulkifli, R.M., Muhamad, I.I., Ya’akob, H., Nur, H., 2017. A review of Acalypha indica L. (Euphorbiaceae) as traditional medicinal plant and its therapeutic potential. J. Ethnopharmacol. 207, 146–173. https://doi.org/10.1016/j.jep.2017.06.019

Zavala-Sánchez, M.A., Pérez-González, C., Arias-García, L., Pérez-Gutiérrez, S., 2009. Anti-inflammatory activity of Wigandia urens and Acalypha alopecuroides. Afr. J. Biotechnol. 8. https://doi.org/10.4314/ajb.v8i21.66071
